# Predation risk shapes the degree of placentation in natural populations of live‐bearing fish

**DOI:** 10.1111/ele.13487

**Published:** 2020-03-12

**Authors:** Andres Hagmayer, Andrew I. Furness, David N. Reznick, Myrthe L. Dekker, Bart J. A. Pollux

**Affiliations:** ^1^ Department of Animal Sciences Wageningen University 6708 WD Wageningen Netherlands; ^2^ Department of Ecology and Evolutionary Biology University of California Irvine CA 92697 USA; ^3^ Department of Biological and Marine Sciences University of Hull HU6 7RX Hull UK; ^4^ Department of Biology University of California Riverside CA 92521 USA

**Keywords:** Life‐history, live‐bearing, matrotrophy, placenta, placentotrophy, Poeciliidae, predation, superfetation, Trexler–DeAngelis, viviparity

## Abstract

The placenta is a complex life‐history trait that is ubiquitous across the tree of life. Theory proposes that the placenta evolves in response to high performance‐demanding conditions by shifting maternal investment from pre‐ to post‐fertilisation, thereby reducing a female’s reproductive burden during pregnancy. We test this hypothesis by studying populations of the fish species *Poeciliopsis retropinna* in Costa Rica. We found substantial variation in the degree of placentation among natural populations associated with predation risk: females from high predation populations had significantly higher degrees of placentation compared to low predation females, while number, size and quality of offspring at birth remained unaffected. Moreover, a higher degree of placentation correlated with a lower reproductive burden and hence likely an improved swimming performance during pregnancy. Our study advances an adaptive explanation for why the placenta evolves by arguing that an increased degree of placentation offers a selective advantage in high predation environments.

## Graphical abstract

The degree of placentation (i.e. Matrotrophy Index) in the placental live‐bearing fish species *Poeciliopsis retropinna* is associated with predation risk. Specifically, females from high predation localities exhibit a significantly higher degree of placentation, compared to females from low predation populations.

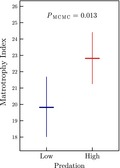

## Introduction

Understanding the origin and elaboration of complex traits is of fundamental interest to evolutionary biologists. Placentas are complex organs that have independently evolved many times throughout the animal kingdom in widely divergent lineages (Wourms 1981; Wooding & Burton 2008; Blackburn 2015; Wake 2015; Ostrovsky *et al.* 2016). This repeated evolution points to a possible adaptive advantage (Losos *et al.* 1998), however, the nature of this advantage remains elusive.

To date, two adaptive hypotheses (termed the resource allocation and locomotor cost hypotheses) provide potential explanations for why the placenta evolves (i.e. its selective advantage). Both hypotheses treat the placenta as a life‐history adaptation that evolves in response to ecological selection pressures (Thibault & Schultz 1978; Trexler & DeAngelis 2003). Furthermore, both hypotheses posit that the ancestral state is for all nutrients to be prepackaged in the form of egg yolk (lecithotrophy) and that the derived state is for nutrients to be supplied to embryos throughout pregnancy via a placenta (placentotrophy). This is presumably achieved via a gradual shift in the timing of nutrient provisioning from pre‐ to post‐fertilisation. If true, then the evolution of the placenta should coincide with a reduction in egg mass at fertilisation without affecting the total investment per neonate at birth (Reznick *et al.* 2002; Trexler & DeAngelis 2003; Pollux *et al.* 2009). The live‐bearing fish family Poeciliidae is a useful system in which to test these ideas, because it contains species that span a continuum of pre‐ versus post‐fertilisation nutrient provisioning, ranging from strictly lecithotrophic to highly placentotrophic. In addition, placentotrophy has evolved independently numerous times in this family (Reznick *et al.* 2002; Pollux *et al.* 2014; Furness *et al.* 2019).

The resource allocation hypothesis (Trexler & DeAngelis 2003) posits that the evolution of the placenta permits females to produce greater brood sizes and hence attain higher fecundity than lecithotrophic females when sufficient resources are available to carry the developing embryos to term. Moreover, placental females rely on a steady nutritional supply to provision embryos throughout pregnancy. Trexler & DeAngelis (2003) therefore argued that the ability to selectively abort embryos and recycle this investment, should resource conditions suddenly deteriorate, is a crucial preadaptation for the evolution of placentation. Studies in the Poeciliidae have not shown that they are able to do this, suggesting that the conditions under which the placenta might be favoured by natural selection are restricted to environments characterised by high and stable resource conditions (Reznick *et al.* 1996; Trexler 1997; Banet & Reznick 2008; Banet *et al.* 2010; Pollux & Reznick 2011).

The locomotor cost hypothesis (Thibault & Schultz 1978; Pollux *et al.* 2009; Pires *et al.* 2011) postulates that the placenta evolves to offset some of the locomotor costs associated with a live‐bearing mode of reproduction. The physical and physiological burden of pregnancy negatively affects a female’s locomotor performance in a broad range of live‐bearing animals (Seigel *et al.* 1987; Plaut 2002; Noren *et al.* 2011; Fleuren *et al.* 2019). The evolution of the placenta should allow females to attain higher fitness, because the production of smaller eggs at fertilisation reduces a female’s reproductive burden during pregnancy. This improves body streamlining and locomotor performance, notably without sacrificing reproductive output. The presumed benefit of a higher degree of placentation is that the improved swimming performance offers a selective advantage to females in performance‐demanding (e.g. high predation) environments because it enhances survival probability (Pollux *et al.* 2009; Pires *et al.* 2011). Whereas the resource allocation hypothesis has already been the focus of several empirical studies (Pires *et al.* 2007; Banet & Reznick 2008; Banet *et al.* 2010; Pollux & Reznick 2011; Bassar *et al.* 2014), the locomotor cost hypothesis has not yet been subject to similar systematic investigation.

Here, we quantify the degree of placentation in high and low predation populations of the placental live‐bearing fish species *Poeciliopsis retropinna* (family Poeciliidae, Regan 1908) in Costa Rica. We test a key prediction of the locomotor cost hypothesis, which is that placentation evolves in performance‐demanding (high predation) environments. If the placenta is favored under high predation conditions, we should find a higher degree of placentation in high predation populations. Theory further predicts that this higher degree of placentation should be due to a smaller egg size at fertilisation without affecting the number, size, or quality of offspring at birth, translating to a lower reproductive burden for females during pregnancy (Thibault & Schultz 1978; Pollux *et al.* 2009; Pires *et al.* 2011). Finally, we discuss the potential fitness advantage of evolving a higher degree of placentation in high predation populations.

## Material and methods

### Study species and collection sites

*Poeciliopsis retropinna* is found in freshwater streams of varying water velocity and predation pressure in Costa Rica and Panama (Bussing 2002). They are observed in habitats with low predation risk (no strongly piscivorous species present), or co‐occurring with one or more piscivorous predator species: *Parachromis dovii*, *Eleotris picta,* and *Gobiomorus maculatus*. During gestation, *P. retropinna* females transfer nutrients to developing embryos via a placenta. The degree of post‐fertilisation maternal provisioning in this species is extensive, with offspring increasing in dry mass more than 100‐fold during gestation (MI = 117) (Reznick *et al.* 2002). Moreover, *P. retropinna* has superfetation, the ability to carry several broods at different developmental stages.

During February and March 2013, 2017, and 2018, *P. retropinna* were collected at 27 different locations in the Rio Terraba and Rio Coto drainages in the province of Puntarenas, Costa Rica (Table S1). Two of the locations were repeatedly sampled resulting in 29 study populations. When water visibility was high (i.e. in all but one location), the occurrence of piscivorous predator species was most effectively detected using underwater visual census by three independent snorkelers. Censuses took place during daytime (*c*. 3–5 h per location/snorkeler) at different positions along each river. In one location (Rio Conte), water visibility was low and predator community was assessed using seine and cast nets. We found 17 ‘high’ predation localities where piscivorous predator species were present, and 12 ‘low’ predation localities where predators were absent (Table S1). At each location, 5–37 adult females were collected, euthanised with an overdose of MS‐222, and preserved in 5% formaldehyde.

### Laboratory measurements

Maternal standard length and the proportion of body fat were measured using established protocols (Supporting Information Methods 1.1). 28 of the 29 sampled populations contained pregnant females (Table S1), therefore all subsequent anatomical and statistical analyses were carried out only with females from 28 populations (*n*_preg_ = 463). The ovaries were dissected to count the total number of embryos (fecundity), regressors (aborted embryos), broods at different developmental stages (superfetation), embryos in a brood (brood size), and to determine the developmental stage and average dry mass of embryos in a brood (Table 1). The developmental stages are based on morphological criteria described in Haynes (1995) and range from 0 (eggs at fertilisation, no development) to 45 (fully developed embryos). Fecundity was calculated by excluding stage 0 embryos, because it was difficult to assess if 0‐staged eggs were fertilised or not. Instead, embryos at developmental stage 2, rather than 0, were defined as ‘eggs at fertilisation’.

**Table 1:** Summary of maternal life‐history traits

Maternal life‐history traits		
Egg mass at fertilisation	Dry mass of eggs at fertilisation (i.e. developmental stage 2)	
Offspring mass at birth	Dry mass of fully developed embryos (i.e. developmental stage 45)	
Proportion egg fat	Egg fat at fertilisation divided by dry mass of eggs at fertilisation	
Proportion offspring fat	Offspring fat at birth divided by offspring dry mass at birth	
Reproductive allotment	Proportion of the mother’s dry mass allocated to reproduction (i.e. embryo dry mass, regressor dry mass, and placental dry mass)	
Absolute reproductive allotment	Total dry mass allocated to reproduction (i.e. embryo dry mass, regressor dry mass, and placental dry mass)
Brood size	Number of embryos in a given brood	
Fecundity	Number of embryos carried by a female counted across all broods excluding stage 0 embryos	
Superfetation	Number of broods at different developmental stages	
Abortion incidence	Number of regressors (i.e. aborted embryos) divided by the sum of the number of regressors and embryos	

### Quantification of the degree of placentation

The Matrotrophy Index (MI), calculated as the ratio of offspring mass at birth to egg mass at fertilisation, was used as an unbiased measure of the degree of placentation (Reznick *et al.* 2002). Some live‐bearing species allocate all resources to eggs prior to fertilisation in the form of large fully‐yolked eggs (termed lecithotrophy). These embryos lose dry mass over the course of gestation due to metabolic processes, leading to an MI < 1. Other species allocate nutrients to the developing offspring post‐fertilisation throughout pregnancy (termed matrotrophy). Such species have an MI > 1, indicating embryos gain dry mass during pregnancy. Placentotrophy represents one specific type of matrotrophy that is achieved through a follicular placenta, roughly an analog to the mammalian placenta (Pollux *et al.* 2009). Because the MI is determined by both egg mass at fertilisation and offspring mass at birth, an increase in the degree of placentation can be brought about by an increase in offspring mass at birth and/or a decrease in egg mass at fertilisation.

The MI for *P. retropinna* was estimated in relation to (1) a given population in a specific year, and (2) high and low predation risk by using the Bayesian programming environment JAGS (Plummer 2003) in R v 3.5 (R Core Team 2019) (Supporting Information Methods 1.2, 1.3).

In (1), ln‐transformed embryo dry mass was fitted as a function of the developmental stage of embryos (stage), stage^2^, proportion of maternal body fat (BF), maternal standard length (SL), and BF × stage. For each population, the model estimates year‐specific intercepts and slopes on stage. This allows for the prediction of MI for a given population in a specific year. In addition, the model includes mother identity as additional intercept to allow for variation among females that is not accounted by maternal BF and SL. The population‐specific MI’s were subsequently calculated by dividing offspring mass at birth (stage 45) by egg mass at fertilisation (stage 2) that were predicted for a given population in a specific year and for a female of overall average SL and BF. Since the MI’s are predicted for a female of the same SL and BF, the resulting MI’s are independent of these traits (Fig. S1).

In (2), ln‐transformed embryo dry mass was fitted as a function of stage, stage^2^, predation, BF, SL, BF × stage and predation × stage. For each population, the model estimates year‐specific intercepts to account for systematic differences among populations within a year. Moreover, the model includes mother identity as additional intercept to allow for variation among females that is not accounted for by maternal BF and SL. The MI for high and low predation females was subsequently calculated by dividing offspring mass at birth by egg mass at fertilisation that were predicted for a given predation regime and for a female of overall average SL and BF.

### Quantification of life‐history variation among predation regimes

The effects of predation on life‐history traits (egg mass at fertilisation, offspring mass at birth, proportion of egg and offspring fat, reproductive allotment, brood size, fecundity, superfetation, and abortion incidence) were analysed by fitting each trait as a function of (1) high and low predation risk, and (2) predator community in a series of (generalised) linear mixed effect models in R v 3.5 (R Core Team 2019), using the package lme4 (Bates *et al.* 2015). Considering *Parachromis dovii* (P), *Eleotris picta* (E)*,* and *Gobiomorus maculatus* (G) as predator species, we observed six different predation categories in nature: low (no strongly piscivorous species present), G, E, EG, PG, and P. Except in the case of reproductive allotment, additional fixed effects included the proportion of maternal body fat (BF) and standard length (SL). Reproductive allotment is defined as the proportion of the mother’s dry mass allocated to reproduction, and hence accounts for female dry mass, rather than BF and SL. Maternal BF predictably responds to experimental manipulation of food availability in the laboratory (Reznick *et al.* 1996; Banet & Reznick 2008; Banet *et al.* 2010; Pollux & Reznick 2011), and is believed to be a good indicator of fish condition (Leips *et al.* 2013). Therefore, accounting for maternal BF may enable us to partly decouple the effects of predation risk and food availability on life‐history traits. The association of life‐history traits with maternal BF and SL is reported and discussed in the Supporting Information (Table S2–S10; Fig. S2). In the case of reproductive allotment, fecundity and superfetation, the developmental stage of the most‐developed brood was fitted as an additional fixed effect to account for females early in the reproductive cycle. Population, year, river, and population × year were fitted as random intercepts accounting for spatio‐temporal non‐independence of observations. Likewise, in the case of brood size, mother identity was fitted as additional random intercept to correct for pseudo‐replication, because brood size is measured multiple times in females with superfetation.

To optimise normality and homoscedasticity of model residuals, reproductive allotment, abortion incidence, proportion of egg and offspring fat and maternal BF were arcsine square‐root transformed. Fecundity, superfetation, and brood size were fitted in generalised linear mixed effect models using a log link for the Poisson‐distributed responses.

### Path analysis

Differences in reproductive allotment (RA) among populations could be due to effects on several life‐history traits. For instance, RA could be decreased by reducing brood size or superfetation, which in turn decreases the number of embryos (fecundity). Alternatively, RA diminishes when producing smaller eggs at fertilisation or offspring at birth. We used a path analysis to determine the contribution of each of these life‐history traits to differences in RA among predation regimes. In total, three (generalised) linear mixed effect models, implemented in MCMCglmm (Hadfield 2010), were used to estimate all paths (Supporting Information Methods 1.4). Each model was refitted as a function of an intercept only (null model) to compare the deviance information criterion (DIC) of the full model against that of the null model (ΔDIC).

In the first model, egg mass at fertilisation, offspring mass at birth, average brood size for a given mother, and superfetation were fitted in a multivariate model as a function of high and low predation risk allowing for the covariance between the residuals of all responses (ΔDIC = −272.75). In the second model, maternal fecundity was fitted as a function of superfetation and average brood size for a given mother, as changes in both brood size and superfetation will affect fecundity (ΔDIC = −388.449). The third model subsequently predicts absolute RA as a function of fecundity, egg mass at fertilisation, and offspring mass at birth (ΔDIC = −135.094).

All three models included the proportion of maternal body fat and standard length as fixed effects. In the case of superfetation and fecundity, the developmental stage of the most‐developed brood was an additional fixed effect (see above). To aid convergence, we did not fit a random year effect (which was effectively zero), but otherwise the random effect structure was the same as above. Furthermore, all continuous input variables were *z*‐standardised to obtain standardised partial regression coefficients (β^*^) that take values between −1 and 1 (Schielzeth 2010). In the case of Poisson‐distributed responses (fecundity and superfetation), β^*^ was obtained retrospectively by dividing the estimated slope (β) by the phenotypic standard deviation of the response variable (σ_Y_). However, σ_Y_ was indirectly estimated using the variance of the predicted model fits on the link scale (σ^2^_log(_ *_Ŷ_*_)_) and the pseudo‐R‐squared (*R* ^2^) (Menard 2011):

$$\sigma_{Y}=\sqrt{\sigma_{\log\left(\hat{Y}\right)}^{2}+R^{2}.}$$

The effect of predation on RA, mediated through a specific maternal life‐history trait, is then given by the direct effect of predation on the life‐history trait and its contribution to the RA. This effect is quantified by multiplying β^*^ of predation on the life‐history trait with β^*^ of the life‐history trait on RA.

## Results

**Figure 1:**
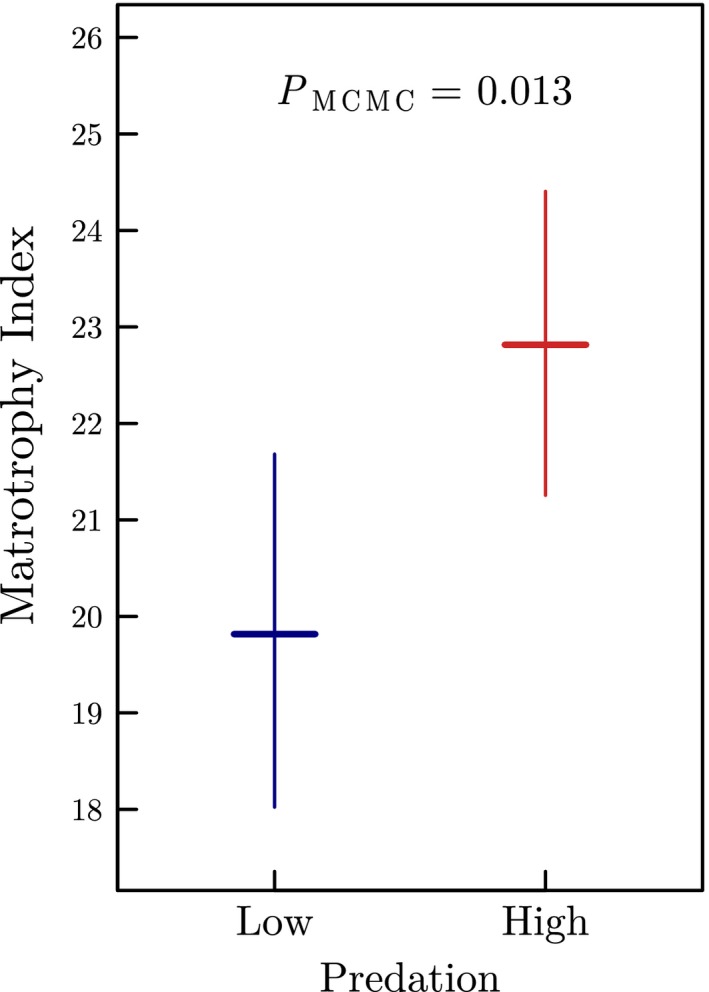
The degree of placentation in *Poeciliopsis retropinna* populations, expressed as the Matrotrophy Index (MI ± 95% posterior density CI), in relation to high and low predation risk (piscivorous predator species present or absent, respectively). The MI is predicted for a female of overall average standard length and proportion of body fat (i.e. body fat = 0.16, standard length = 53 mm). The posterior Bayesian *P*‐value (*P*_MCMC_) is given at the top.

There was a more than two‐fold range in the estimated degree of placentation among the 28 natural populations of *P. retropinna* (MI ranging from 14.87 to 32.34; Table S1). Consistent with the locomotor cost hypothesis, we found that *P. retropinna* females from high predation (HP) localities exhibit a significantly higher degree of placentation (MI_HP_: mean = 22.86, 95% CI = 21.30–24.46), compared to females from low predation (LP) populations (MI_LP_: mean = 19.86, 95% CI = 18.06–21.73; *P*_MCMC_ = 0.013; Fig. 1; Fig. S3). This lends compelling support to the idea that predation risk may drive the evolution of placentas at a micro‐evolutionary level.

**Figure 2:**
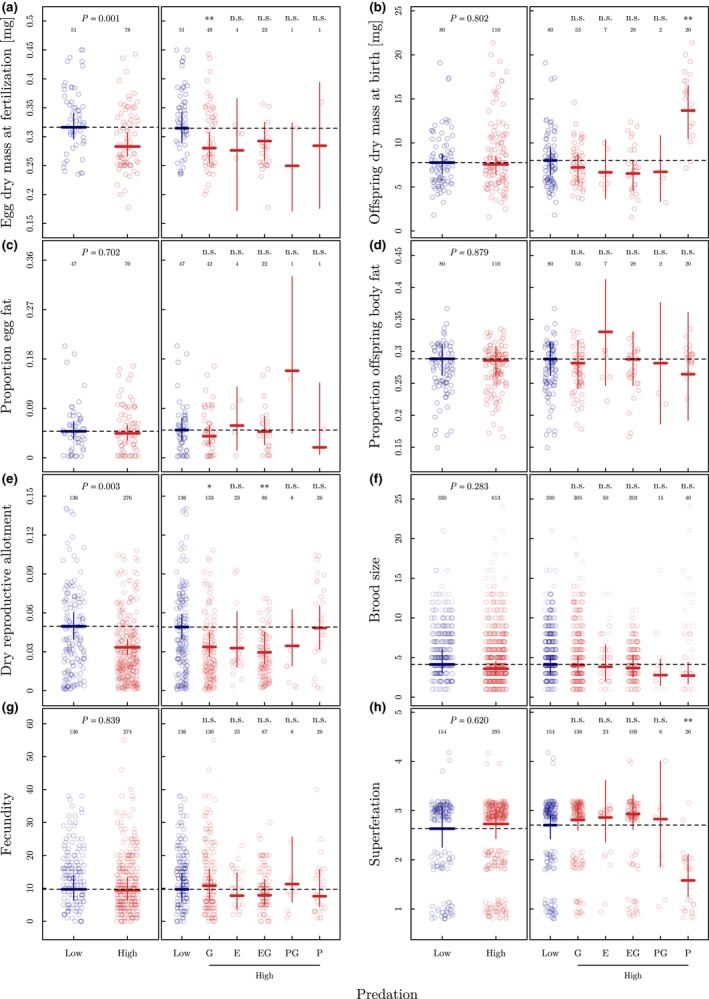
Life‐history characteristics of *Poeciliopsis retropinna* in relation to predation risk. (a) Egg dry mass at fertilisation (developmental stage 2), (b) Offspring dry mass at birth (developmental stage 45), (c) Proportion egg fat at fertilisation, (d) Proportion offspring fat at birth, (e) dry reproductive allotment, (f) brood size, (g) maternal fecundity (number embryos in all broods combined), and (h) degree of superfetation (± 95% CI) as a function of high and low predation risk (piscivorous predator species present or absent; left panels) and predator community (G: *Gobiomorus maculatus*; E: *Eleotris picta*; P: *Parachromis dovii*; right panels) estimated in the models described in Table S2–S9, S22–S29. Except in (e), all model predictions account for the proportion of maternal body fat and maternal standard length, which are kept constant at the overall population mean (i.e. body fat = 0.16, standard length = 53 mm). In (e) and (g–h), the developmental stage of the most‐developed brood carried by the female is kept constant at the overall median (i.e. developmental stage 42.5). Data points (red: high predation; blue: low predation) correspond to the ‘jittered’ raw data. Sample size and *P*‐value are given at the top of each panel. Significant codes: *P* < 0.001***, < 0.01**, ≤ 0.05*, > 0.05 n.s.

However, to test the validity of the locomotor cost hypothesis, at least three additional subpredictions have to be evaluated. First, the higher degree of placentation in HP populations should not be correlated with systematic differences in environmental conditions, because then other local environmental conditions account (at least partly) for the observed interpopulation variation in the degree of placentation, either by influencing egg mass at fertilisation or offspring mass at birth. We measured several water quality parameters at each location (salinity, water velocity, hardness, NH_4_^+^, PO_4_^3‐^, and dissolved oxygen) (Supporting Information Methods 1.5), but did not find any clear differences between HP and LP populations (Table S11–S16; Fig. S4). Moreover, when interpopulation variation in placentation is directly related to predation risk and the water quality parameters, we still found a higher degree of placentation in HP populations (Supporting Information Methods 1.6; Fig. S5). Second, the higher degree of placentation of HP females should not be associated with a difference in maternal traits such as proportion of body fat (BF) and standard length (SL), as they are known to influence the degree of placentation (Hagmayer *et al.* 2018). This subprediction is met, as the degree of placentation of HP and LP females is predicted for a female of the same SL and BF. In addition, comparisons of maternal traits did not reveal any difference in maternal SL (*t*_19.078 _= −0.353, *P* = 0.728; Table S17) or BF (*t*_5.315_ = 0.395, *P* = 0.708; Table S18) between HP and LP populations (Fig. S6). Third, the higher degree of placentation of HP females should be due to the production of smaller eggs at fertilisation. The locomotor cost hypothesis predicts that an increase in the degree of placentation will convey a potential adaptive benefit to females only if the higher MI is the consequence of producing smaller eggs at fertilisation, while offspring size and number at birth, as well as the degree of superfetation remain unchanged. Consistent with these predictions, we found that independent of maternal BF and SL, HP females displayed an average 11% reduction in egg dry mass at fertilisation compared to LP females (*t*_109.112_ = −3.316, *P* = 0.001; Table S2; Fig. 2a), while offspring dry mass at birth did not differ between HP and LP females (*t*_20.752_ = −0.253, *P* = 0.802; Table S3; Fig. 2b). Likewise, the proportion of egg fat at fertilisation (*t*_43.513_ = −0.386, *P* = 0.702; Table S4; Fig. 2c), proportion of offspring fat at birth (*t*_20.866_ = −0.153, *P* = 0.879; Table S5; Fig. 2d) and the degree of superfetation (*z* = 0.496, *P* = 0.620; Table S6; Fig. 2h) did not differ between HP and LP females.

This raises the question how an increase in the degree of placentation in HP populations could confer a potential adaptive advantage to females during pregnancy? If all the above criteria are met, a higher degree of placentation should result in a lower reproductive allotment (RA) during pregnancy, notably without sacrificing fecundity. In line with this, we found that HP females had dry RA’s that were on average 33% smaller than those of LP females (*t*_21.342_ = −3.290, *P* = 0.003; Table S7; Fig. 2e), while brood size (*z* = −1.074, *P* = 0.283; Table S8; Fig. 2f) and fecundity (*z* = −0.203, *P* = 0.839; Table S9; Fig. 2g) did not differ between HP and LP females. Notably, the observed reduction in dry RA by 33% (17.9 mg) equals the dry mass of 2.6 offspring at birth (stage 45) that are produced by a female of average dry mass (Supporting Information Methods 1.7; Table S19; Fig. S7). Moreover, we found that the observed maternal traits and life‐history pattern in HP females are independent of the type of predator community (Table S20–S29; Fig. 2,S6). This suggests that different piscivorous predator species drive similar life‐history adaptations in *P. retropinna*.

**Figure 3:**
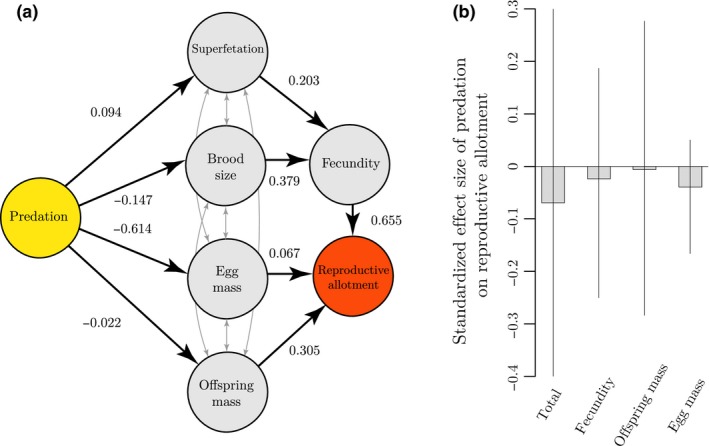
(a) Graphical illustration of the relationships between predation, egg dry mass at fertilisation (i.e. developmental stage 2), offspring dry mass at birth (i.e. developmental stage 45), brood size, fecundity, superfetation, and absolute dry reproductive allotment. The direction of the arrows represents the directionality of the relationships. The numbers equal standardised partial regression coefficients (i.e. the strength of the relationships), which take values between −1 and 1. Grey arrows represent the covariances between the residuals of the responses measured on the same observational unit. (b) Standardised effect size of predation on the reproductive allotment mediated through the maternal life‐history traits (± 95% CI). These values equal the contribution of each of the maternal life‐history traits to differences in the reproductive allotment among predation regimes. The paths via superfetation and brood size are summed together, as both are mediated via fecundity. Note that (a) and (b) show that the total effect of predation on the reduction of reproductive allotment is mainly mediated through egg mass at fertilisation.

Finally, the standardised effect sizes (β^*^) confirm that the reduction in RA in HP females is mainly mediated through egg mass at fertilisation (β^*^ = −0.040), rather than offspring mass at birth (β^*^ = −0.006), or brood size, superfetation, and maternal fecundity (β^*^ = −0.024) (Fig. 3). In other words, the seemingly small difference in egg mass at fertilisation of 11% largely contributes to the observed difference in RA of 33%. Collectively, these findings show that females from HP localities have a higher degree of placentation and that this significantly reduces their reproductive burden during pregnancy, without negatively affecting their reproductive output.

## Discussion

### The degree of placentation in natural populations is shaped by predation

Comparisons of the degree of placentation among natural populations are of particular interest, because they may provide insights into the evolutionary processes that drive the elaboration of placentas at a macro‐evolutionary level (Hansen & Martins 1996; Arnold *et al.* 2001; Reznick & Ricklefs 2009; Rolland *et al.* 2018). To date, few studies have evaluated interpopulation variation in the degree of placentation and these did not examine (or used insufficient replicates to make reliable inferences about) the ecological factors that may have driven differences in the degree of placentation among populations (e.g. Schrader & Travis 2005, compared two populations of the highly placental *Heterandria formosa*; Pires *et al.* 2007, two populations of the highly placental *Poeciliopsis prolifica*; Gorini‐Pacheco, Zandonà & Mazzoni 2017, three populations of the moderate placental *Phalloceros harpagos*).

Here, we show that predation risk in streams correlates with the degree of placentation among 28 natural populations of the highly placental *P. retropinna*. Specifically, female *P. retropinna* that co‐occur with piscivorous predator species produce smaller eggs at fertilisation, while giving birth to an equal number, size, and quality of offspring. The life‐history adjustments lead to a significantly higher MI, and hence degree of placentation, in HP females (MI_HP_ = 22.86) compared to LP females (MI_LP_ = 19.86).

Our results show that the production of smaller eggs at fertilisation, associated with an increase in the degree of placentation in HP populations, results in a lower reproductive allotment (RA) for females during pregnancy. Interestingly, comparative studies in the fish families Poeciliidae and Zenarchopteridae show that placentation is consistently associated with a lower RA (Reznick *et al.* 2007; Bassar *et al.* 2014). In this study, we show that similar patterns are found at an intra‐specific level, among natural populations of a placental species. This suggests that the association between (higher degrees of) placentation and a lower RA may be a general feature of the evolution of placentas, both at a micro‐ and macro‐evolutionary level.

The locomotor cost hypothesis proposes that a lower RA may offer an adaptive benefit to pregnant live‐bearing females, because it leads to a more slender body shape and hence improved swimming performance. A number of recent studies seem to support this idea. Fleuren *et al. *(2018) showed that placental species are more slender at the beginning of pregnancy compared to non‐placental ones and that, consistent with the locomotor cost hypothesis (Pollux *et al.* 2009), this morphological advantage of the placenta diminishes over the course of gestation. Superfetation may amplify this morphological advantage (Pollux *et al.* 2009) (Fig. S8). A lower RA and streamlined body shape are associated with a reduced drag on the female body (Quicazan‐Rubio *et al.* 2019), higher sustained swimming performance (Plaut 2002), improved fast‐start escape response (Ghalambor *et al.* 2004; Fleuren *et al.* 2019), and enhanced survival probability (Walker *et al.* 2005; Plath *et al.* 2011; Laidlaw *et al.* 2014).

The increased degree of placentation and associated decreased RA in HP populations may thus lead to an enhanced predator escape performance. The question is: what is the resulting ‘survival benefit’ of HP females compared to LP females? In an attempt to quantify the survival advantage of a HP versus a LP female *P. retropinna*, we used estimates of the effects of body shape on locomotor performance in *Poeciliopsis turneri* (Fleuren *et al.* 2019) and the probability of evading a predator strike in *Poecilia reticulata* (Walker *et al.* 2005). The difference in wet reproductive allotment between HP and LP female *P. retropinna* is largest just before the parturition of a brood (210.31 mg; Table S30; Fig. S8). Relative to body size (0.0013 SL^−3^), this difference is equivalent to an improvement in maximum escape velocity of 1.12 SL s^−1^ in *P. turneri* (Fleuren *et al.* 2019). Based on *P. reticulata*, such an improvement in escape performance would translate to an increased chance of successfully evading a predator strike of 1.2% (Walker *et al.* 2005) (Supporting Information Methods 1.8; Fig. S8). At first glance, this may not seem large, however, the strength of selection needed to explain observed rates of evolution can be extremely weak (Lande 1976). If we assume that, (1) the degree of placentation of HP and LP females represent two different genotypes, (2) the HP genotype has a survival advantage of 1.2%, (3) there is no mixing of genotypes, and (4) the HP genotype begins with a frequency of 1% in the population, then it only takes 625 generations (or 469 years based on the generation time of 0.75 years in *P. retropinna*) to make up 95% of the population (Supporting Information Methods 1.9; Fig. S9). The maximum time available for the evolution of extensive placentation in the genus *Poeciliopsis* is estimated to be 0.75–2.36 million years (Reznick *et al.* 2002). Nevertheless, care must be taken when translating the estimates of the effects of body shape on locomotor performance and survival probability based on other poeciliid species to *P. retropinna*. Although the relationship between body shape and escape performance was shown to not differ between three different placental poeciliid species (Fleuren *et al.* 2019), the relationship between locomotor performance and survival probability is based on *P. reticulata*. Compared to *P. retropinna*, *P. reticulata* is non‐placental without superfetation which may affect the association with survival also other than via improving locomotor performance (e.g. behavioural differences).

To be considered an adaptation, differences in placentation among HP and LP populations must be heritable, rather than entirely phenotypically plastic. Turcotte *et al. *(2008) have investigated the patterns of maternal provisioning in naturally occurring hybrids between *Poeciliopsis monacha*, a lecithotrophic species that produces large eggs at fertilisation, and *P. lucida*, a moderately matrotrophic species that produces small eggs and provisions embryos with nutrients throughout gestation. Hybrids produced intermediate‐sized eggs, suggesting that egg mass at fertilisation is to some degree genetically controlled. Given that egg mass is heritable and subject to selection, the observed differences in egg mass at fertilisation in response to predation risk are thus likely to be evolved.

In conclusion, our results provide the first compelling evidence in support of the locomotor cost hypothesis by showing that an increase in the degree of placentation can offer a selective advantage in high ‘performance‐demanding’ (i.e. high‐predation localities) environments (Pollux *et al.* 2009; Pires *et al.* 2011).

### No support for the resource allocation hypothesis

The resource allocation hypothesis proposes that the placenta evolved because the associated reduction in egg size at fertilisation allows females to attain higher fitness through increased brood sizes. Trexler & DeAngelis (2003) argued that the ability to selectively abort embryos and recycle this investment, should resource conditions suddenly deteriorate, is a crucial preadaptation for the evolution of placentation and that female fat reserves might serve to buffer placental species from fluctuating resource conditions.

Two findings appear to tentatively argue against this hypothesis. First, prior studies in four independent placental lineages have shown that poeciliid fish are not able to abort embryos in response to sudden adverse resource conditions (Reznick *et al.* 1996; Banet & Reznick 2008; Banet *et al.* 2010; Pollux & Reznick 2011). Second, if female fat storage serves to buffer placental species from fluctuating resource conditions, then abortion incidence should increase when food conditions become unfavorable and maternal fat reserves insufficient to fully buffer females during gestation. In other words, better‐conditioned females (those carrying larger fat reserves) should be less likely to abort embryos. Contrary to this expectation, we found an increase in the abortion incidence with a higher proportion of maternal body fat: better‐conditioned females were more likely to abort embryos (*t*_230.335_ = 2.186, *P* = 0.03; Table S10; Fig. S2). The mechanisms behind this higher rate of embryo abortion in better‐conditioned female *P. retropinna* are currently unclear and require further investigation.

### Conclusion: placentation facilitates the evolution of new life‐history opportunities

To date, the effect of predation risk on poeciliid life history has almost exclusively been studied in non‐placental species including *Poecilia reticulata* (Reznick & Endler 1982; Reznick *et al.* 1990; Reznick & Bryga 1996), *Brachyrhaphis rhabdophora* (Johnson & Belk 2001), *Brachyrhaphis episcopi* (Jennions *et al.* 2006)*, Gambusia hubbsi* (Downhower *et al.* 2000)*,* and *Xiphophorus hellerii* (Basolo & Wagner 2004). As predicted by theoretical studies (Gadgil & Bossert 1970; Law 1979; Michod 1979), guppies adaptively respond to increased adult mortality by devoting a larger percentage of their body mass to their developing offspring (Reznick & Endler 1982; Reznick *et al.* 1990). Furthermore, they exhibit shorter time intervals between successive broods and produce more, but smaller, offspring (Reznick & Endler 1982; Reznick 1982a, 1983; Reznick *et al.* 1990). These predation‐driven life‐history differences were shown to have a genetic basis (Reznick 1982b). Smaller offspring, however, have a lower ability to capture prey (Lankheet *et al.* 2016), compete for food (Bashey 2006), escape predators (Gibb *et al.* 2006), and hence have a lower survival probability in low food and/or high predation environments (Bashey 2006; Dial *et al.* 2016). Thus, it has been argued that selection for increased maternal fecundity and smaller offspring in Trinidadian guppies indicates that maternal fitness, rather than offspring fitness, may have dominated in shaping the evolution of offspring size in response to predation (Dial *et al.* 2016).

Our study reveals that matrotrophic species may be able to adopt strikingly different solutions to deal with high predation, solutions that are not possible in lecithotrophic species such as the guppy. In high predation environments, females of the placental *P. retropinna* reduced their reproductive allotment, notably without reducing the size or number of offspring at birth. This is likely to be advantageous for the mother, as the decreased reproductive allotment improves her locomotor performance (Plaut 2002; Ghalambor *et al.* 2004; Fleuren *et al.* 2019; Quicazan‐Rubio *et al.* 2019) and survival probability in high predation environments (Plath *et al.* 2011; Laidlaw *et al.* 2014). This life‐history adaptation is however unavailable to lecithotrophic species. In theory, lecithotrophic species could respond to high predation risk by (1) reducing the reproductive allotment at the expense of either fecundity or offspring size, or (2) increasing fecundity at the expense of maternal mortality. Studies have shown that lecithotrophic species principally do the latter (Reznick & Endler 1982; Reznick *et al.* 1990; Reznick & Bryga 1996; Downhower *et al.* 2000; Johnson & Belk 2001; Basolo & Wagner 2004; Jennions *et al.* 2006). The evolution of the placenta opens up novel opportunities for females to respond to high predation risk that are not available to lecithotrophic species. Specifically, placental females are able to reduce their reproductive allotment seemingly without any reproductive cost; i.e. without sacrificing either fecundity or offspring quality at birth.

## Competing interest statement

None declared.

## Authorship

AIF and BJAP conceived the project idea. BJAP obtained funding and directed the project. AH, AIF, MLD and BJAP planned the fieldwork and collected the data. AH and AIF carried out the dissections. AH analysed the data. AH wrote the first draft of the manuscript supervised by AIF and BJAP. DNR and MLD critically reviewed the initial manuscript and provided helpful input. All authors approved the final manuscript.

## Supporting information

Supplementary Material

## Data Availability

The data that support the findings of this study are available from Dryad Digital Repository (https://doi.org/10.5061/dryad.cvdncjt16).
