# Supplementary material for: Predation risk shapes the degree of placentation in natural populations of live‐bearing fish
Source: Ecol Lett. 2020 Mar 12;23(5):831–40. doi: 10.1111/ele.13487 (PMC7187176; doi:10.1111/ele.13487)
Supplement: Supplementary file 1 — Supplementary Material [file ELE-23-831-s001.pdf]

Supporting Information for

Predation risk shapes the degree of placentation in natural populations of live-bearing fish

Andres Hagmayer, Andrew I. Furness, David N. Reznick, Myrthe L. Dekker, and Bart J. A. Pollux

1 Supporting Methods

1.1 Laboratory measurements

The fish samples were transported to the Reznick lab (University of California Riverside, USA) and Pollux lab (Wageningen University, the Netherlands) for anatomical dissections. The standard length of preserved specimens was measured to the nearest mm from the tip of the upper jaw to the outer margin of the hypural plate, using a caliper. Female dry mass was measured to the nearest 0.01 mg on a Mettler Toledo AE163 Microbalance (specimens collected in 2013) or Mettler Toledo XP205 delta range (specimens collected in 2017 and 2018) (Mettler Instruments Corp., Hightstown, New Jersey, USA) after removing the ovary and air-drying the female overnight at 60°C in a drying oven. Female lean mass was measured by extracting the fat twice with anhydrous diethyl ether (Fisher Scientific) to remove triglycerides, and by subsequently air-drying and re-weighting the female (see above). The proportion of maternal body fat was then calculated by subtracting maternal lean mass from maternal dry mass divided by maternal dry mass. The embryo dry mass for a given brood was calculated as the dry mass of the brood, measured to the nearest 0.01 mg after air-drying overnight at 60°C (see above), divided by the number of embryos in the brood [9].

1.2 Population-specific Matrotrophy Indices

Population-specific Matrotrophy Indices (MI's) were estimated using the Bayesian programming environment `JAGS` [8]. For this, ln-transformed embryo dry mass was fitted in a linear model as a function of the developmental stage of embryos (second order polynomial), the proportion of maternal body fat, maternal standard length, and the interaction between the developmental stage of embryos and the proportion of maternal body fat. For each population, the model estimates year-specific intercepts and year-specific slopes on the developmental stage of the embryos (second order polynomial). This allows to predict the MI for a given population in a specific year. In addition, the model includes the mother identity as additional intercept to account for variation among females that is not accounted by maternal body fat and standard length. We used N(0,5^2) priors for the proportion of maternal body fat, maternal standard length, and the interaction between the developmental stage of embryos and the proportion of maternal body fat. For mother identity, we used a N(0,σ^2) prior with the common variance $\sigma^2 \sim$ Inv-Gamma(0.01, 0.01), parameterized with shape and rate parameters. The year-specific intercepts and year-specific slopes on the developmental stage of the embryos (second order polynomial) were estimated using N(μ, σ^2) priors, with the common mean $\mu \sim$ N(0,2^2) and the common standard deviation $\sigma \sim$ Student-$t_{(0,\infty)}(0, 1^2, 1)$, parameterized with mean, variance, and degrees of freedom.

The population-specific MI's were subsequently calculated by dividing offspring dry mass at birth (developmental stage 45) by egg dry mass at fertilization (developmental stage 2) that were predicted for a given population in a specific year and for a female of overall average standard length (i.e., $\bar{y}_{\text{standard length}}$=53 mm) and proportion of body fat (i.e., $\bar{y}_{\text{body fat}}$=0.16). Since all population-specific MI's are predicted for a female of the same length and body fat, the resulting MI's are independent of these traits (Fig. S1). However, the 'raw' MI for each population in a specific year (i.e., predicted based on the observed, rather than the average maternal body fat and standard length) is also provided (Table S1).

Three MCMC chains were run for 1,500,000 iterations, with a burnin of 500,000 and a thinning of 1000. Convergence was assessed by visual examination of the traces and by checking that $\hat{R} < 1.01$.

The model fit was assessed using a posterior predictive check on the predictions of embryo mass.

$$\log(\overline{\mathrm{EM}}_{i,j}) \sim N(\alpha_{\mathrm{pop}\times\mathrm{year}} + \alpha_{\mathrm{mother}} + \beta_{1,\mathrm{pop}\times\mathrm{year}}\mathrm{stage}_{i,j} + \beta_{2,\mathrm{pop}\times\mathrm{year}}\mathrm{stage}^2_{i,j} + \beta_3\mathrm{mBF}_i + \beta_4\mathrm{mSL}_i + \beta_5\mathrm{mBF}_i \times \mathrm{stage}_{i,j}, \sigma^2), \tag{1}$$

where $\log(\overline{\mathrm{EM}}_{i,j})$ corresponds to the ln-transformed mean embryo dry mass of the jth brood carried by the ith female. $\alpha_{\mathrm{pop}\times\mathrm{year}}$ corresponds to the year-specific intercept of a given population and α_{mother} to the mother identity. $\beta_{1,\mathrm{pop}\times\mathrm{year}}\mathrm{stage}_{j,i}$ is the year-specific slope of a given population on the developmental stage of the jth brood carried by the ith female (second order polynomial), mBF_i the arcsin square-root transformed proportion of body fat of the ith female, mSL_i the standard length of the ith female, and σ the residual standard deviation.

1.3 Predation-specific Matrotrophy Indices

Predation-specific Matrotrophy Indices (MI's) were estimated using the Bayesian programming environment `JAGS` [8]. For this, ln-transformed embryo dry mass was fitted in a linear model as a function of the developmental stage of embryos (second order polynomial), high and low predation risk (i.e., piscivorous predator species present or absent), the proportion of maternal body fat, maternal standard length, the interaction between the developmental stage of embryos and the proportion of maternal body fat, and the interaction between the developmental stage of embryos and predation risk. For each population, the model estimates an additional year-specific intercept to correct for differences between populations in a specific year. Moreover, the model includes the mother identity as additional intercept to account for variation among females that is not accounted by maternal body fat and standard length. We used N(0,5^2) priors for all fixed effects. For mother identity, we used a N(0,σ^2) prior with the common variance $\sigma^2 \sim$ Inv-Gamma(0.01, 0.01), parameterized with shape and rate parameters. The year-specific intercepts and year-specific slopes on the developmental stage of the embryos (second order polynomial) were estimated using N(μ,σ^2) priors, with the common mean $\mu \sim$ N(0,1^2) and the common standard deviation $\sigma \sim$ Student-$t_{(0,\infty)}(0, 1^2, 1)$, parameterized with mean, variance, and degrees of freedom.
The MI for high and low predation females was subsequently calculated by dividing offspring dry mass at birth (developmental stage 45) by egg dry mass at fertilization (developmental stage 2) that were predicted for a given predation regime (i.e., piscivorous predator species present or absent) and for a female of overall average standard length (i.e., $\overline{y}_{\text{standard length}}$=53 mm) and proportion of body fat (i.e., $\overline{y}_{\text{body fat}}$=0.16).
Three MCMC chains were run for 1,500,000 iterations, with a burnin of 500,000 and a thinning of 1000. Convergence was assessed by visual examination of the traces and by checking that $\hat{R} < 1.01$.
The model fit was assessed using a posterior predictive check on the predictions of embryo mass.

$$\log(\overline{\mathrm{EM}}_{i,j}) \sim N(\alpha_{\mathrm{pop}\times\mathrm{year}} + \alpha_{\mathrm{mother}} + \mathrm{intercept} + \beta_1\mathrm{stage}_{i,j} + \beta_2\mathrm{stage}^2_{i,j} + \beta_3\mathrm{predation}_i + \beta_4\mathrm{mBF}_i + \beta_5\mathrm{mSL}_i + \beta_6\mathrm{mBF}_i \times \mathrm{stage}_{i,j} + \beta_7\mathrm{predation}_i \times \mathrm{stage}_{i,j}, \sigma^2), \tag{2}$$

where $\log(\overline{\mathrm{EM}}_{i,j})$ corresponds to the ln-transformed mean embryo dry mass of the jth brood carried by the ith female. $\alpha_{\mathrm{pop}\times\mathrm{year}}$ corresponds to the year-specific intercept of a given population and α_{mother} to the mother identity. intercept is the overall intercept, $\beta_1\mathrm{stage}_{j,i}$ the developmental stage of the jth brood carried by the ith female (second order polynomial), $\mathrm{predation}_i$ the predation risk experienced by the ith female, mBF_i the arcsin square-root transformed proportion of body fat of the ith female, mSL_i the standard length of the ith female, and σ the residual standard deviation.

1.4 Path analysis

Differences in reproductive allotment among populations could be due to effects on several life-history traits. For instance, reproductive allotment could be decreased by reducing brood size or superfetation, which in turn decreases the number of embryos (i.e., fecundity). Alternatively, reproductive allotment diminishes when producing smaller eggs at fertilization or offspring at birth. We used a path analysis to determine the contribution of each of these life-history traits to differences in reproductive allotment among predation regimes. In total, three (generalized) linear mixed effect models implemented in `MCMCglmm` [3] were used to estimate all paths.

In the first model, we estimated the direct effect of predation risk on z-standardized egg dry mass at fertilization (developmental stage 2) (ze), z-standardized offspring dry mass at birth (developmental stage 45) (zo), z-standardized average brood size for a given mother (zb), and the degree of superfetation (s) in a multivariate model:

$$[ze, zo, zb, s] \sim \mu + \mathbf{X}\beta + \mathbf{Z}r, \tag{3}$$

where μ is the intercept vector, β the vector of fixed effects, $\mathbf{X}$ the corresponding design matrix, and $\mathbf{Z}$ is a design matrix for additional random terms r. z-standardized egg dry mass at fertilization, offspring dry mass at birth, and the average brood size for a given mother are assumed to be Gaussian-distributed. Superfetation was formulated in a generalized linear mixed model framework using a log link for the Poisson-distributed response. Fixed effects include predation risk (i.e., piscivorous predator species present or absent), z-standardized proportion of maternal body fat, z-standardized maternal standard length, and the z-standardized developmental stage of the most-developed brood in the case of superfetation. Besides an error, the random terms include population and river identity accounting for spatio-temporal non-independence of observations. Moreover, the interaction between population identity and year quantifies variation of a given population between years. The multivariate framework allows for the covariance between the residuals of all responses. As priors, we used the default normal priors for the fixed effects with the expected value of 0 and variance 10^{12}, and inverse-Wishart priors for the variances with the expected value of 1 and degree of belief of 3.002. The number of iterations was 5,500,000, with a burnin of 500,000 and a thinning of 5000.

In the second model, maternal fecundity was fitted as a function of superfetation and average brood size for a given mother, as changes in both brood size and superfetation will affect the number of embryos:

$$f_i \sim Po(\lambda_i), \log(\lambda_i) = \alpha + \alpha_{\text{pop}} + \alpha_{\text{river}} + \alpha_{\text{pop}\times\text{year}} + \\ \beta_1 zs_i + \beta_2 zb_i + \beta_3 \text{mBF}_i + \beta_4 \text{mBF}_i^2 + \beta_5 \text{mSL}_i + \beta_6 \text{latestStage}_i), \tag{4}$$

where f_i corresponds to the fecundity (i.e., number embryos) of the ith female. α corresponds to the overall intercept, and α_{pop}, α_{river}, and $\alpha_{\text{pop}\times\text{year}}$ to the random intercepts. zs_i is the z-standardized degree of superfetation, zb_i the z-standardized average brood size, $z\text{mBF}_i$ the z-standardized proportion of maternal body fat (second order polynomial), $z\text{mSL}_i$ the z-standardized maternal standard length, and $z\text{latestStage}_i$ the z-standardized developmental stage of the most developed brood of the ith female. As priors, we used the default normal priors for the fixed effects with the expected value of 0 and variance 10^{12}, and inverse-Wishart priors for the variances with the expected value of 1 and degree of belief of 0.002. The number of iterations was 1,500,000, with a burnin of 500,000 and a thinning of 1000.

The third model subsequently predicts the absolute dry reproductive allotment as a function of fecundity, egg dry mass at fertilization, and offspring dry mass at birth:

$$z\text{ra}_i \sim N(\alpha + \alpha_{\text{pop}} + \alpha_{\text{river}} + \alpha_{\text{pop}\times\text{year}} + \\ \beta_1 zf_i + \beta_2 ze_i + \beta_3 zo_i + \beta_4 z\text{mBF}_i + \beta_5 z\text{mSL}_i, \sigma^2), \tag{5}$$

where $z\text{ra}_i$ corresponds to the absolute dry reproductive allotment of the ith female. α corresponds to the overall intercept, and α_{pop}, α_{river}, and $\alpha_{\text{pop}\times\text{year}}$ to the random intercepts. zf_i is the z-standardized maternal fecundity (i.e., number embryos), ze_i the z-standardized egg dry mass at fertilization (developmental stage 2), zo_i the z-standardized offspring dry mass at birth (developmental stage 45), $z\text{mBF}_i$ the z-standardized proportion of maternal body fat, $z\text{mSL}_i$ the z-standardized maternal standard length of the ith female, and σ the residual standard deviation. As priors, we used the default normal priors for the fixed effects with the expected value of 0 and variance 10^{12}, and inverse-Wishart priors for the variances with the expected value of 1 and degree of belief of 0.002. The number of iterations was 1,500,000, with a burnin of 500,000 and a thinning of 1000.
For all models, convergence was assessed by visual examination of the traces and by checking that the autocorrelations of the parameter chain was less than 0.1. In addition, each model was re-fitted as a function of an intercept only (null model) to compare the deviance information criterion (DIC) of the full model against the DIC of the null model (ΔDIC).

1.5 Measurements of water parameters

The water velocity was measured at each location to the nearest 0.01 m$\cdot$ s^{-1} with a Höntzsch Vane Wheel FA current meter (type ZS30 GFE md20 T/100-2/p10, Höntzsch Instruments, Waiblingen, Germany). Depending on the uniformity of the flow, the water velocity was taken at 9—17 incremental observation points across a transect of the stream. At each observation point, the mean water velocity was defined as the average of three repeated measurements at a height above the stream bed equal to 0.4 times the depth at that location. When the water depth exceeded 60 cm, the mean water velocity

was calculated as the average between the velocities measured at 0.2 times the water depth and 0.8 times the water depth [5]. Each location was additionally characterized by measuring salinity (S) by using the ExStik II pH/conductivity/TDS meter (Extech Instruments, Nashua, USA), hardness ($mg\cdot L^{-1}$) with a titrimetric color-test kit (Merck KGaA, Darmstadt, Germany), ammonium concentration (NH_4^+) ($mg\cdot L^{-1}$) as a proxy for the nitrogen loading of a stream by using a colorimetric ammonium test (Merck KGaA, Darmstadt, Germany), phosphate concentration (PO_4^{3-}) ($mg\cdot L^{-1}$), as phosphorus is an important determinant of primary production in freshwater ecosystems [12] by using a colorimetric phosphate test (Merck KGaA, Darmstadt, Germany), and dissolved oxygen (%) with the ExStik DO600 meter (Extech Instruments, Nashua, USA). Salinity and dissolved oxygen were measured 1—3 times at each location, whereas hardness, ammonium concentration, and phosphate concentration were measured only once.

Salinity was then predicted for each location at 25°C in the following linear model by Maximum Likelihood:

$$\mathrm{S}_{i,j} \sim N(\alpha + \beta_1 \mathrm{location}_i + \beta_2 \mathrm{temperature}_{i,j}, \sigma^2), \tag{6}$$

where $\mathrm{S}_{i,j}$ corresponds to the jth measurement of salinity at location i, α to the overall intercept, $\mathrm{location}_i$ to the ith location, $\mathrm{temperature}_{i,j}$ to the jth measurement of water temperature at location i, and σ to the residual standard deviation.

Dissolved oxygen was predicted for each location at 13:00 hours and 25°C in the following linear model by Maximum Likelihood:

$$\mathrm{oxygen}_{i,j} \sim N(\alpha + \beta_1 \mathrm{location}_i + \beta_2 \mathrm{temperature}_{i,j} + \beta_3 \mathrm{time}_{i,j} + \beta_4 \mathrm{time}^2_{i,j}, \sigma^2), \tag{7}$$

where $\mathrm{oxygen}_{i,j}$ corresponds to the jth measurement of dissolved oxygen at location i, α to the overall intercept, $\mathrm{location}_i$ to the ith location, $\mathrm{temperature}_{i,j}$ to the jth measurement of water temperature at location i, $\mathrm{time}_{i,j}$ to the z-standardized numeric day time of the jth measurement of dissolved oxygen at location i, and σ to the residual standard deviation.

1.6 Relationship between population-specific Matrotrophy Indices and environmental variables

Either by influencing egg mass at fertilization or offspring mass at birth, many population-specific factors are likely to contribute to the observed interpopulation variation in the degree of placentation. To quantify the potential effects of additional water parameters at each location on the degree of placentation, the population-specific Matrotrophy Indices (MI's) estimated in the model described in Equation 1 were fitted as a function of all measured environmental variables (predation risk, salinity, water velocity, hardness, NH_4^+, PO_4^{3-}, and dissolved oxygen) in a linear mixed effect model using Restricted Maximum Likelihood. The continuous predictors were z-standardized in order to make them comparable [11]. Moreover, the model includes river identity as additional intercept to account for variation among rivers that is not accounted by the measured environmental parameters.

$$
\begin{aligned}
z\mathrm{MI}_i \sim N(&\alpha_{\mathrm{river}} + \mathrm{intercept} + \\
&\beta_1 \mathrm{predation}_i + \beta_2 z\mathrm{salinity}_i + \beta_3 z\mathrm{hardness}_i + \\
&\beta_4 z\mathrm{water\ velocity}_i + \beta_5 z\mathrm{NH}^{+}_{4,i} + \beta_6 z\mathrm{PO}^{3-}_{4,i} + \beta_7 z\mathrm{O}_{2,i}, \sigma^2),
\end{aligned}
\tag{8}
$$

where $z\mathrm{MI}_i$ corresponds to the Matrotrophy Index for the ith population in a specific year estimated in the model described in Equation 1. α_{river} corresponds to the river-specific intercept and intercept to the the overall intercept. $\mathrm{predation}_i$ is the predation risk (i.e., piscivorous predator species present or absent), $z\mathrm{salinity}_i$ the z-standardized water salinity (S), $z\mathrm{hardness}_i$ the z-standardized hardness ($\mathrm{mg \cdot L^{-1}}$), $z\mathrm{water\ velocity}_i$ the z-standardized mean water velocity ($\mathrm{m \cdot s^{-1}}$), $z\mathrm{NH}^{+}_{4,i}$ the z-standardized ammonium concentration ($\mathrm{mg \cdot L^{-1}}$), $z\mathrm{PO}^{3-}_{4,i}$ the z-standardized phosphate concentration ($\mathrm{mg \cdot L^{-1}}$), $z\mathrm{O}_{2,i}$ the z-standardized dissolved oxygen (%), and σ the residual standard deviation.

1.7 Embryo growth during gestation

Embryo growth during gestation was estimated as the exponential relationship between embryo dry mass and the developmental stage of embryos (second order polynomial) in a linear mixed effect model by Restricted Maximum Likelihood [4]. The model included maternal dry mass, and the interaction between the developmental stage of embryos and maternal dry mass as additional fixed effects (Fig. S7).

Mother identity was fitted as random intercept to correct for pseudo-replication. Population, year, and river identity were fitted as random intercepts accounting for spatio-temporal non-independence of observations. Moreover, the interaction between population identity and year was fitted as random intercept to quantify the variation of a given population between years:

$$
\begin{aligned}
\log(\overline{\mathrm{EM}}_{i,j}) \sim N(&\alpha + \alpha_{\mathrm{mother}} + \alpha_{\mathrm{pop}} + \alpha_{\mathrm{year}} + \alpha_{\mathrm{river}} + \alpha_{\mathrm{pop \times year}} + \\
&\beta_1 \mathrm{stage}_{i,j} + \beta_2 \mathrm{stage}^2_{i,j} + \beta_3 \mathrm{mM}_i + \beta_4 \mathrm{mM}_i \times \mathrm{stage}_{i,j}, \sigma^2),
\end{aligned}
\tag{9}
$$

where $\log(\overline{\mathrm{EM}}_{i,j})$ corresponds to the ln-transformed mean embryo dry mass of the jth brood carried by the ith female. α corresponds to the overall intercept, α_{mother}, α_{pop}, α_{year}, α_{river}, and $\alpha_{\mathrm{pop \times year}}$ to the random intercepts. $\mathrm{stage}_{i,j}$ is the developmental stage of the jth brood carried by the ith female, mM_i the dry mass of the ith female, and σ the residual standard deviation.

1.8 Relationship between reproductive allotment, locomotor performance, and survival probability

Fleuren et al. 2019 [2] studied the locomotor performance in three placental live-bearing fish species (family Poeciliidae) that exhibit different levels of superfetation. Particularly, they used computer-vision based techniques to study changes in body shape (e.g. volume) and three-dimensional fast-start escape performance (e.g. maximum escape velocity) during pregnancy in *Poeciliopsis turneri*, *Heterandria formosa*, and *Phalloptychus januarius*. The slope between body shape and escape performance was not significantly different between the three species. Here we used the relationship between female volume and maximum escape velocity in *Poeciliopsis turneri* to predict the maximum escape

velocity in *Poeciliopsis retropinna*. *P. retropinna* and *P. turneri* are similar regarding their degree of post-fertilization maternal provisioning and degree of superfetation. We derived female volume from female wet mass, assuming an uniform tissue density (ρ) of 1 g$\cdot$cm^{-3} [10]. The relationship between female volume and maximum escape velocity is then given by:

$$\hat{\mathrm{V}}_{\mathrm{max}} = \alpha + \beta_1 \frac{\mathrm{M}}{\rho \mathrm{SL}^3} + \beta_2 \mathrm{CR}_{\mathrm{max}} \tag{10}$$

where $\hat{\mathrm{V}}_{\mathrm{max}}$ corresponds to the normalized maximum escape velocity (normalized for standard length SL), α to the overall intercept, and $\frac{\mathrm{M}}{\rho \mathrm{SL}^3}$ to the normalized female volume (normalized for standard length SL^3) derived from the tissue density ρ, female wet mass M, and the average standard length of a pregnant *P. retropinna* female. $\mathrm{CR}_{\mathrm{max}}$ is the maximum caudal peduncle curvature rate in the kinematic stage 2 of the fast-start escape response.

The estimated maximum escape velocity for a given wet mass was then used to predict the probability of evading the strike of a natural predator. Based on a study by Walker et al. 2005 [15] with Guppies (*Poecilia reticulata*), the probability of evading a predator (ω) was predicted using four parameters: (1) the initial distance between predator and prey (D_{pred}), (2) the average speed of the predator ($\overline{\nu}_{\mathrm{pred}}$), (3) the evasion path of the prey relative to the strike path of the predator (θ_{pred}), and (4) the maximum velocity of the prey (vmax=$\hat{\mathrm{V}}_{\mathrm{max}} \cdot \mathrm{SL}_{\mathrm{Guppy}}$) estimated in Equation 10:

$$\mathrm{logit}(\omega) = \alpha + \beta_1 \mathrm{vmax} + \beta_2 \theta_{\mathrm{pred}} + \beta_3 \overline{\nu}_{\mathrm{pred}} + \beta_4 D_{\mathrm{pred}} \tag{11}$$

1.9 Simulating selection

To illustrate that the strength of selection needed to explain observed rates of evolution, assuming that genetic drift is not involved, can be extremely weak, we have simulated the frequency p^2 of a high predation genotype (A_1A_1), and the complementary frequency q^2 of a low predation genotype (A_2A_2) over time (i.e., generations). The HP genotype is assumed to have a survival advantage (s) of 1.2%, and there is no mixing of the genotypes. The initial frequency (p_0^2) of the advantageous genotype in the population is assumed to be 0.01.

The initial genotype frequencies are given by:

$$p_0^2, \quad \text{and} \quad q_0^2 = 1 - p_0^2 \tag{12}$$

The fitness ω of the genotypes A_1A_1 and A_2A_2 are given by:

$$\omega_{11} = 1, \quad \text{and} \quad \omega_{22} = 1 - s \tag{13}$$

The mean fitness ϖ of all the individuals in the population at time t is then given by:

$$\varpi_t = p_t^2\omega_{11} + q_t^2\omega_{22} \tag{14}$$

The genotype frequencies after selection can then be calculated by:

$$p_t'^2 = \frac{p_t^2\omega_{11}}{\varpi_t}, \quad \text{and} \quad q_t'^2 = \frac{q_t^2\omega_{22}}{\varpi_t} \tag{15}$$

2 Supporting Results

2.1 Relationship between maternal traits and life-history

Both the proportion of maternal body fat and standard length are significantly associated with maternal fecundity (Table S9). The correlation between maternal fecundity and standard length is strongly positive (z=16.993, P<0.001; Fig. S2). The linear and quadratic relationship between fecundity and body fat are both negative (linear: z=-5.506, P<0.001; Fig. S2; quadratic: z=-4.474, P<0.001). Similarly, brood size shows a negative correlation with proportion of maternal body fat (z=-4.701, P<0.001; Table S8; Fig. S2) and a strong positive correlation with standard length (z=15.713, P<0.001; Table S8; Fig. S2). By contrast, the degree of superfetation is not significantly correlated with the proportion of maternal body fat (z=-1.712, P=0.087; Table S6; Fig. S2) or standard length (z=-0.483, P=0.629; Table S6; Fig. S2). In addition, egg dry mass at fertilization (mg) increases as a function of increased maternal standard length ($t_{117.432}$=4.008, P<0.001; Table S2; Fig. S2), but is not correlated with the proportion of maternal body fat ($t_{116.329}$=-1.270, P=0.207; Table S2; Fig. S2). Offspring dry mass at birth (mg) is positively associated with the proportion of maternal body fat ($t_{79.502}$=4.208, P<0.001; Table S3; Fig. S2) and standard length ($t_{132.520}$=2.191, P=0.030; Table S3; Fig. S2). Abortion incidence shows a negative linear ($t_{213.123}$=-3.615, P<0.001; Table S10; Fig. S2) and a positive quadratic association with standard length ($t_{391.638}$=2.873, P=0.004; Table S10; Fig. S2). Moreover, abortion incidence is positively correlated with the proportion of maternal body fat ($t_{230.335}$=2.186, P=0.03; Table S10; Fig. S2).

3 Supporting Discussion

3.1 Relationship between maternal traits and life-history

Maternal traits can profoundly influence the offspring phenotype and maternal life-history [7]. Consistent with previous findings in *P. retropinna* [4], larger females have greater fecundity, produce larger eggs at fertilization, and offspring at birth (Fig. S2). Furthermore, females that have more fat reserves produce fewer but larger offspring at birth, without investing more in egg size at fertilization. The negative correlation between the proportion of maternal body fat and fecundity may reflect a trade-off between offspring size and number; the production of large offspring may necessarily entail the production of fewer offspring owing to the limited size of the female body cavity [14].

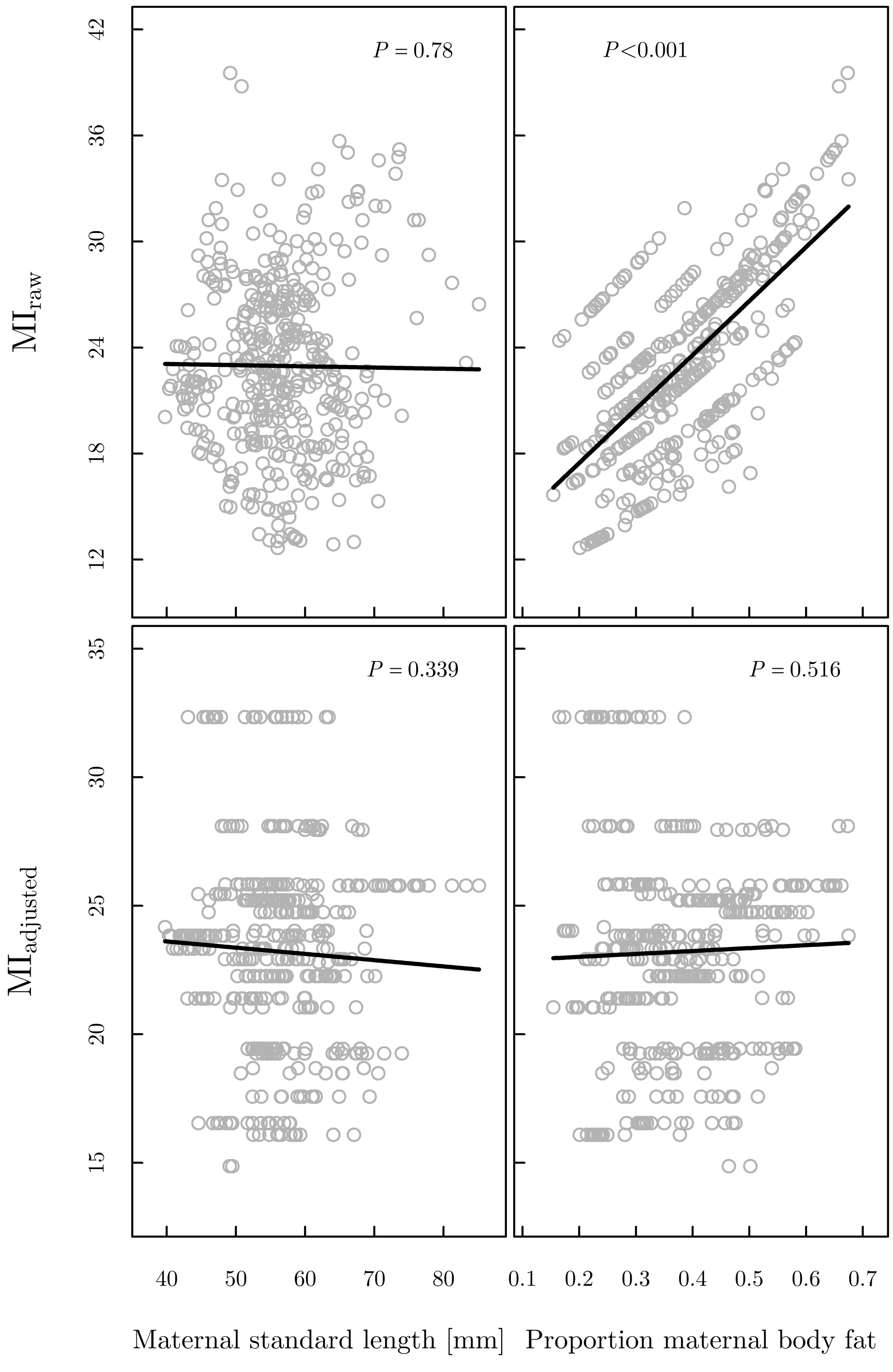

Fig. S1. **Association of the 'raw' Matrotrophy Indices (MI's) (upper panels) and 'adjusted' MI's (lower panels) estimated in the model described in Equation 1 with maternal standard length (mm) (left panels) and proportion of maternal body fat (right panels). The 'raw' MI's are predicted for a given population in a specific year and for the observed values of maternal standard length and body fat, and hence, are not independent of these traits. As it was shown in Hagmayer et al. (2018), the MI is positively associated with maternal body fat, but does not correlate with maternal standard length. By contrast, the 'adjusted' MI's are predicted for a given population in a specific year and for a female of overall average standard length and proportion of body fat. Since the 'adjusted' MI's are predicted for a female of the same length and body fat, the resulting MI's are independent of these traits. *P*-value is given at the top.**

.

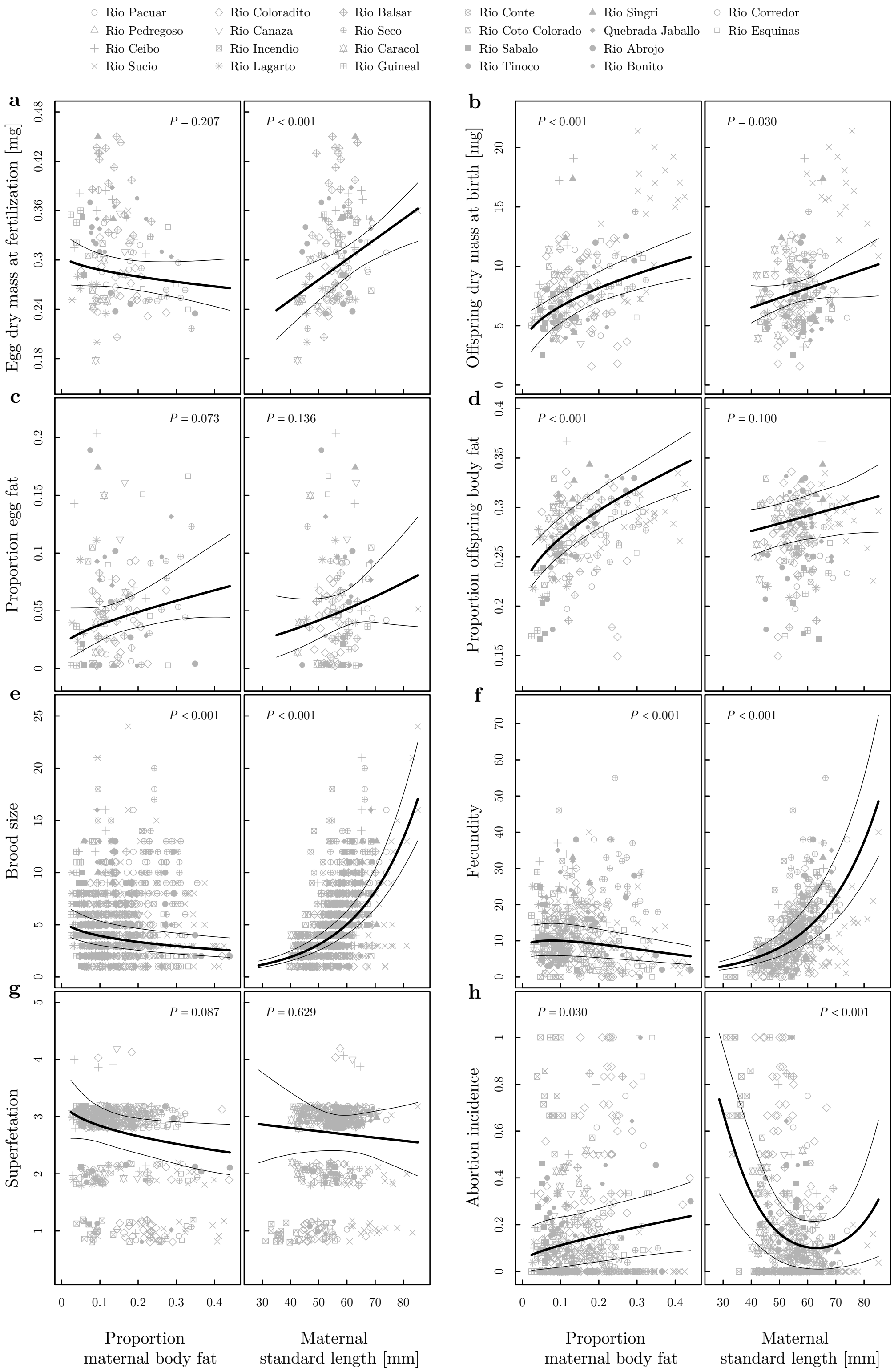

Fig. S2. Life-history characteristics of *Poeciliopsis retropinna* in relation to maternal body fat and size. a Egg dry mass at fertilization (i.e., developmental stage 2) (*n*=129), b offspring dry mass at birth (i.e., developmental stage 45) (*n*=190), c proportion of egg fat at fertilization (i.e., developmental stage 2) (*n*=117), d proportion of offspring fat at birth (i.e., developmental stage 45) (*n*=190), e brood size (*n*=943), f maternal fecundity (i.e., number embryos) (*n*=411), g degree of superfetation (*n*=449), and h abortion incidence (*n*=463) (± 95% CI) as a function of the proportion of maternal body fat (left panels) and standard length (right panels) estimated in the models described in Table S2—-S10. The models are predicted for a high predation female and account for maternal standard length (left panels) and the proportion of maternal body fat (right panels), which are kept constant at the overall population mean (i.e., $\overline{y}_{\textbf{body fat}}$=0.16, $\overline{y}_{\textbf{standard length}}$=53 mm). In f and g, the developmental stage of the most-developed brood carried by the female is kept constant at the overall median (i.e., developmental stage 42.5). Data points correspond to the river-specific raw data. *P*-value is given at the top.

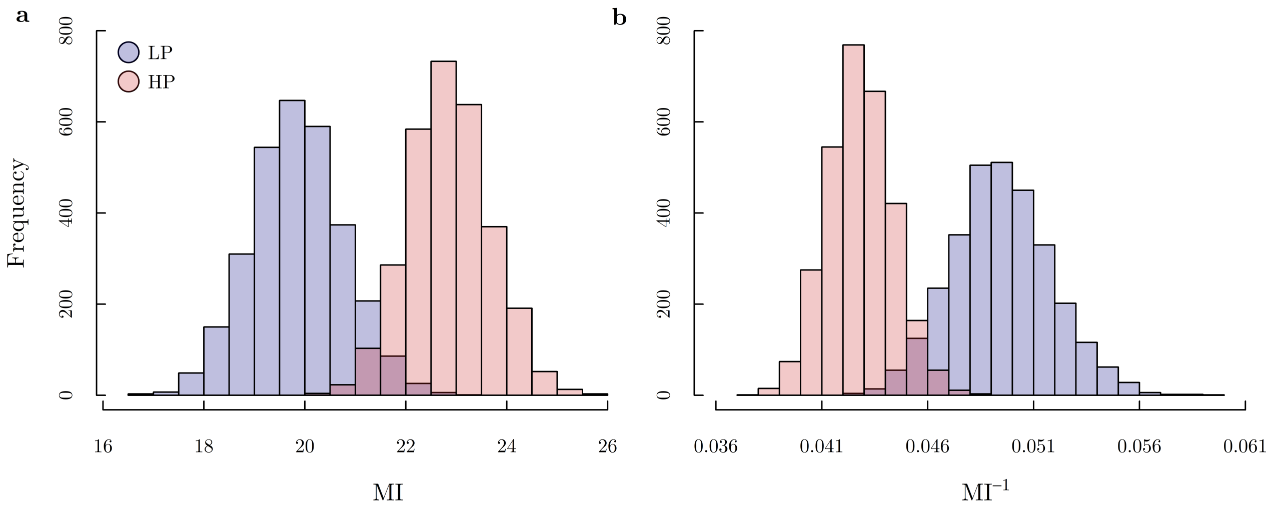

Fig. S3. a Frequency distribution of the posterior estimates of the Matrotrophy Indices (MI's) estimated in the model described in Equation 2. The MI's are predicted for a given predation regime (HP: piscivorous predator species present; LP: piscivorous predator species absent) and for a female of overall average standard length (i.e., $\overline{y}_{\text{standard length}}$=53 mm) and proportion of body fat (i.e., $\overline{y}_{\text{body fat}}$=0.16). b Frequency distribution of the posterior estimates of the inverse MI's (i.e., calculated by dividing egg dry mass at fertilization by offspring mass at birth) to show that all MCMC samples are < 1.

.

The potential mechanisms behind the increased post-fertilization maternal provisioning by larger and better-conditioned females, however, is unclear [4]. By contrast, maternal fecundity is proposed to increase with female size, as a consequence of more space available in the female's body cavity [13]. The physical constraint of the body cavity is also displayed by the populations where *P. retropinna* co-occur with *P. dovii* as the only predator species. Independently of the proportion of maternal body fat and standard length, these females produce very large offspring at birth (Fig. 2), but carry significantly fewer broods at different developmental stages (i.e., superfetation; Fig. 2). These females were mainly collected from a single stream (Rio Sucio) with exceptional high nitrogen loading (NH_4^+ concentration measured in 2017 2.71 $\times$ larger than average), and probably nutrient-rich water. As a result, the females are very large (Table S20; Fig. S6), contain a large amount of body fat (Table S21; Fig. S6), and invest more in offspring size at birth (Fig. 2). Here, we additionally show that the degree of superfetation does not correlate with the proportion of maternal body fat or size in *P. retropinna* (Table S6; Fig. S2).

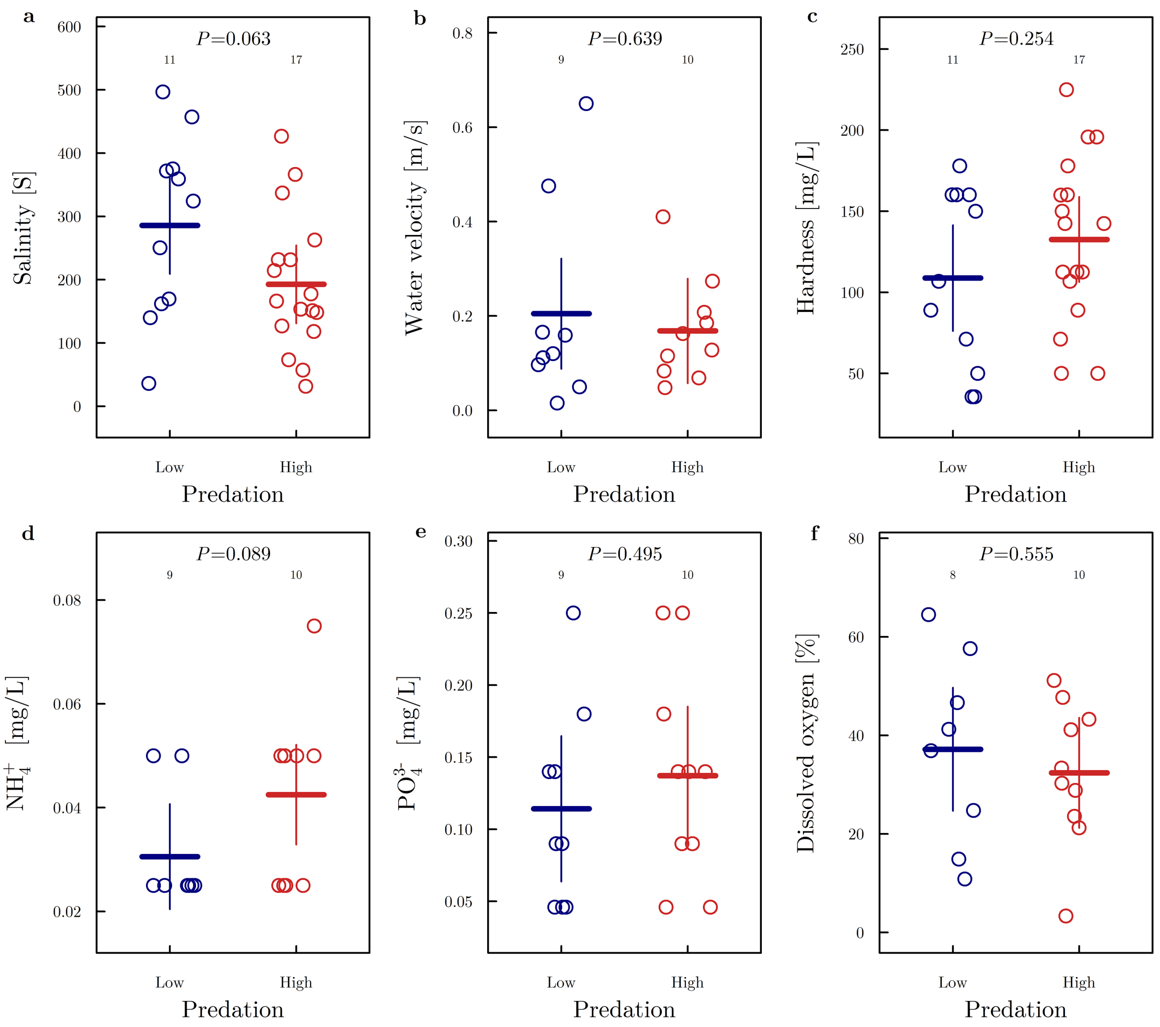

Fig. S4. **Water quality parameters as a function of high and low predation risk (i.e., piscivorous predator species present or absent) estimated in the models described in Table S11—-S16. a Salinity, b mean water velocity, c hardness, d ammonium concentration (NH_4^+), e phosphate concentration (PO_4^{3-}), and f dissolved oxygen (± 95% CI). Data points (red: high predation; blue: low predation) correspond to the 'jittered' raw data. Sample size and *P*-value are given at the top. These data tentatively suggest that there are no obviously large differences in water quality parameters between study locations with and without predators. However, these results should be interpreted with care, because salinity and dissolved oxygen were measured only 1—3 times at each location, and hardness, ammonium concentration, and phosphate concentration were measured even only once. Water quality parameters are likely to vary during the day and throughout the year. A single or only a few measurements per location taken at different times during the day, on different days, and even in different years are therefore unlikely to accurately reflect the yearly mean local abiotic conditions experienced by the fish in each population.**

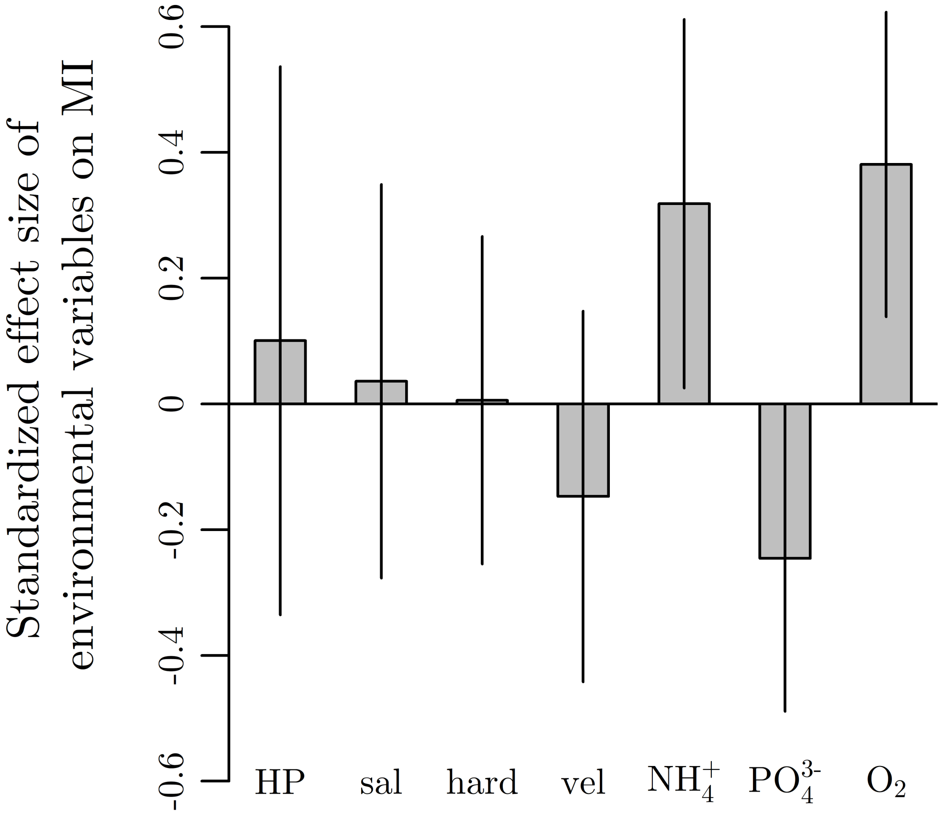

Fig. S5. **Standardized partial regression coefficients (β^*) ($\pm$ se) for the measured environmental variables on the population-specific Matrotrophy Indices (MI's) estimated in the model described in Equation 8. The population-specific Matrotrophy Indices (MI's) were estimated in the model described in Equation 1. The environmental variables include predation risk (HP: high predation, i.e., piscivorous predator species present), salinity (S), hardness (hard), mean water velocity (vel), ammonium concentration (NH_4^+), phosphate concentration (PO_4^{3-}), and dissolved oxygen (O_2). The β^*'s are equivalent to standardized effect sizes that take values between -1 and 1 [11]. Please note that salinity and dissolved oxygen were measured only 1—3 times at each location, and hardness, ammonium concentration, and phosphate concentration were measured even only once. The water quality parameters are likely to vary during the day and throughout the year. A single or only a few measurements per location taken at different times during the day, on different days, and even in different years are therefore unlikely to accurately reflect the yearly mean local abiotic conditions experienced by the fish in each population. Thus, care must be taken when interpreting the relationship between the MI and the water quality parameters. Still, these data show that independently of the measured water quality parameters, we still predict a higher degree of placentation in high predation populations.**

.

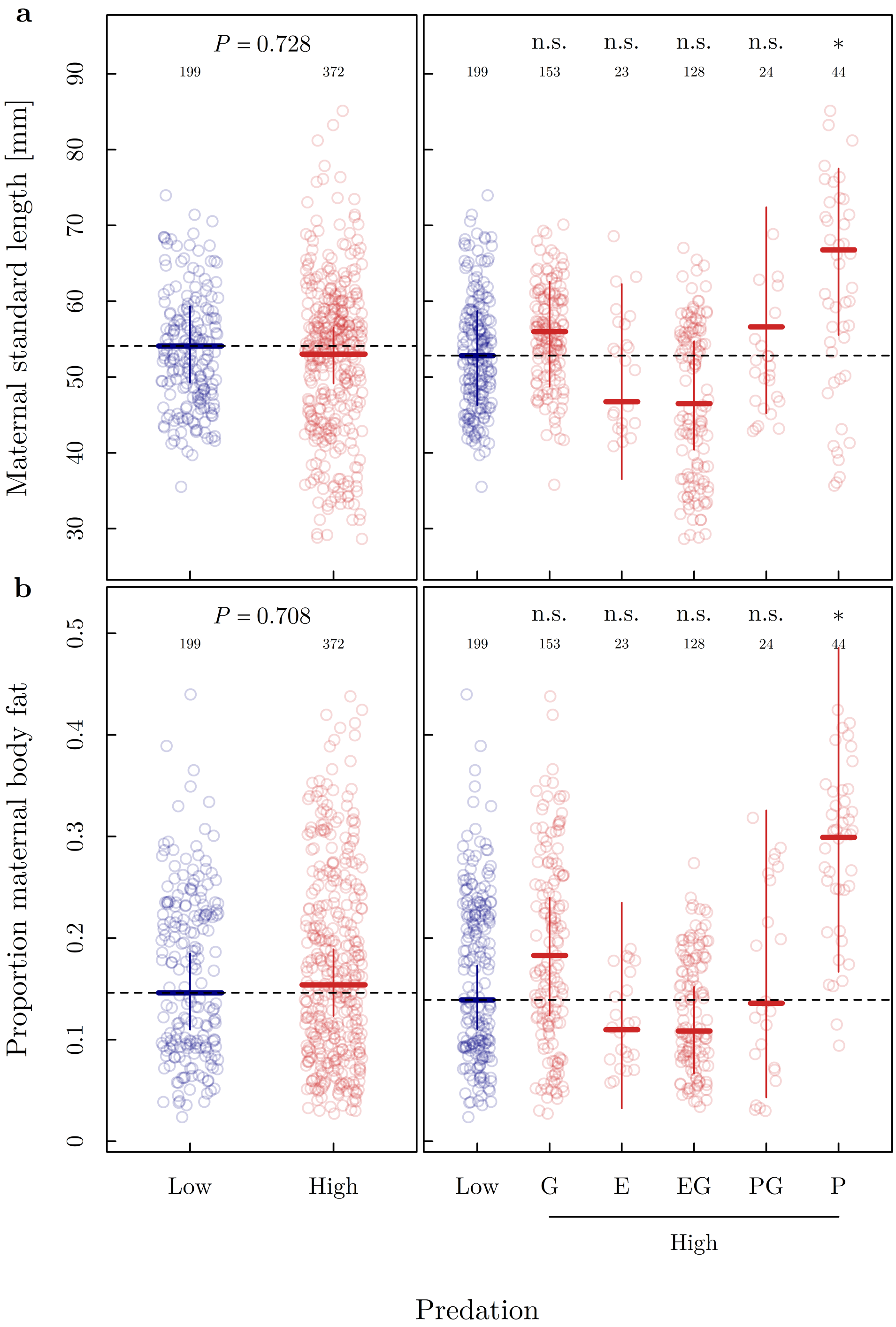

Fig. S6. **a Maternal standard length and b proportion of maternal body fat (± 95% CI) as a function of predation risk estimated in the models described in Table S17—S18,S20—-S21. The model predictions account for the proportion of maternal body fat in a and maternal standard length in b that are kept constant at the overall population mean (i.e., $\overline{y}_{\text{body fat}}$=0.16, $\overline{y}_{\text{standard length}}$=53 mm). left panels: high and low predation risk (i.e., piscivorous predator species present or absent); right panels: predator community. G: *Gobiomorus maculatus*; E: *Eleotris picta*; P: *Parachromis dovii*. Data points (red: high predation; blue: low predation) correspond to the 'jittered' raw data. Sample size and *P*-value are given at the top. Significant codes: *P*$< 0.001^{***}, < 0.01^{**}, \leq 0.05^{*}, > 0.05$ n.s.**

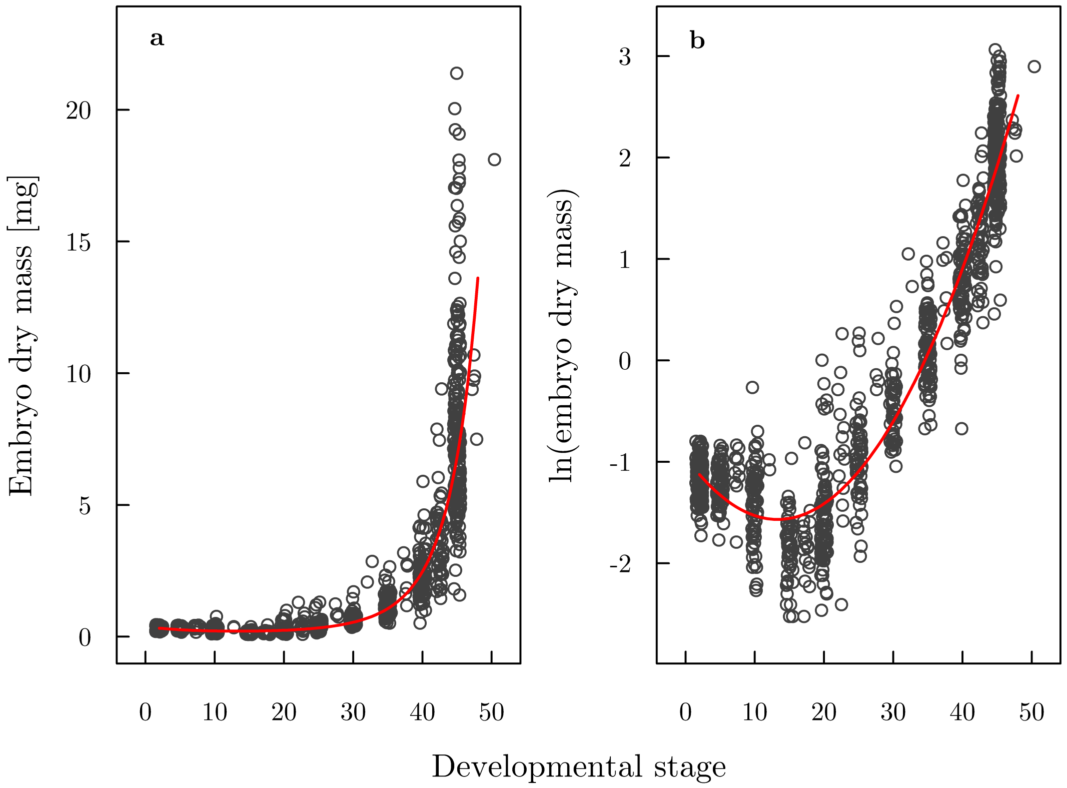

Fig. S7. a Embryo dry mass [mg] and b ln-transformed embryo dry mass as a function of the developmental stage of embryos (2—50) estimated in the model described in Equation 9. The model prediction (red) accounts for maternal dry mass that is kept constant at the overall population mean (i.e., $\overline{y}_{\textbf{dry mass}}$=0.982 g). Data points (dark grey) correspond to the 'jittered' raw data. Please note that embryo dry mass decreases until developmental stage 13. The placenta might not be fully functional during early embryo development. Thus, the mass loss as a result of metabolic processes may outweigh the mass gain as a result of placental provisioning early in pregnancy.

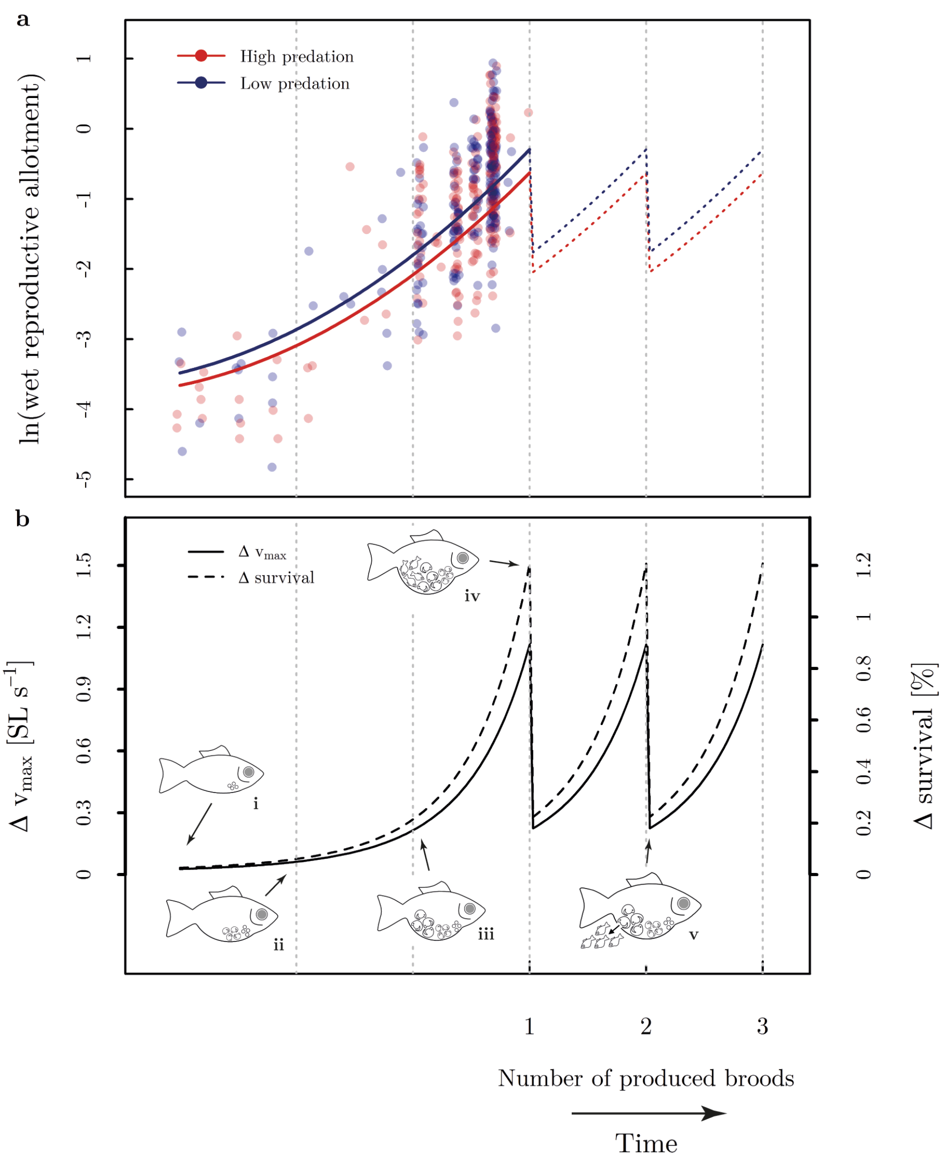

Fig. S8. a ln-transformed absolute wet reproductive allotment as a function of time and high (red) and low (blue) predation risk (i.e., piscivorous predator species present or absent) in *P. retropinna* estimated in the model described in Table S30. Data points correspond to the 'jittered' raw data. The developmental stage of the most developed brood was used to define the time from fertilization to parturition of the first brood (thick solid line). Continuous brood production (i.e. superfetation) subsequently leads to a repetitive pattern of frequent parturition (thin dotted line). Note that high predation females produce smaller eggs at fertilization, which contributes to the decreased wet reproductive allotment at fertilization. Despite producing the same size and number of offspring, superfetation prevents the difference in reproductive allotment at fertilization from becoming zero towards the end of gestation. The difference in reproductive allotment between high and low predation populations is largest before the parturition of a brood (dotted grey lines). b predicted difference in maximum escape velocity (SL$\cdot s^{-1}$; solid line) and survival probability (dotted line) between high and low predation females based on *Poeciliopsis turneri* [2], and *Poecilia reticulata* [15], respectively. i Female early in pregnancy carrying a single brood at developmental stage 2 (i.e., eggs at fertilization); ii female fertilizes a second brood; most-developed brood at developmental stage 18; iii female fertilizes a third brood; intermediate-developed brood at developmental stage 18; most-developed brood at developmental stage 34; iv least-developed brood at developmental stage 18; intermediate-developed brood at developmental stage 34; most-developed brood ready to get born (i.e., stage 50); v female fertilizes a third brood; intermediate-developed brood at developmental stage 18; most-developed brood at developmental stage 34.

.

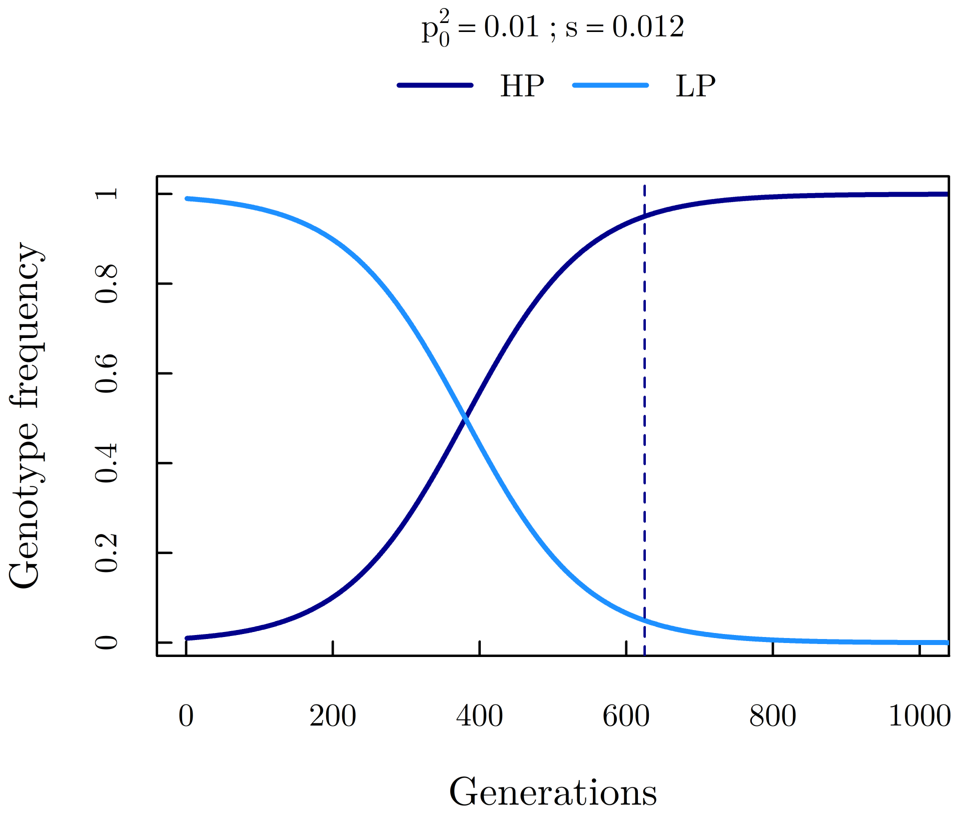

Fig. S9. **Simulated frequency of a high (HP) and low (LP) predation genotype over time (i.e., generations). The HP genotype is assumed to have a survival advantage of 1.2%, and there is no mixing of the genotypes. The initial frequency (p_0^2) of the advantageous genotype in the population is assumed to be 0.01. The dotted line corresponds to the point in time, at which the advantageous genotype makes up 95% of the population.**

.

Table S1. **Sampling locations (plus coordinates), sampling dates, predation risk (HP: piscivorous predator species present; LP: piscivorous predator species absent), number of collected (*n*) and pregnant (n_{preg}) females at each location, and population-specific Matrotrophy Indices (MI's). The MI's are estimated using the Bayesian programming environment `JAGS` [8] and are predicted for a given population in a specific year and for a female of overall average standard length and proportion of body fat. The $\mathrm{MI_{raw}}$'s are predicted for the observed, rather than the average maternal body fat and standard length.**

Location	River	Predation	Coordinates	Sampling date	*n*	n_{preg}	MI	$\mathrm{MI_{raw}}$
Canaza	Rio Canaza	HP	N 08° 39', W 83° 11'	29.03.2013	15	2	22.80	22.70
Ceibo downstream	Rio Ceibo	HP	N 09° 09', W 83° 22'	26.03.2013	24	8	24.02	21.52
Coloradito downstream	Rio Coloradito	HP	N 08° 36', W 82° 54'	28.03.2013	26	24	28.10	27.42
Incendio	Rio Incendio	HP	N 08° 27', W 82° 60'	29.03.2013	27	17	24.17	20.07
Lagarto	Rio Lagarto	HP	N 08° 41' W 83° 05'	30.03.2013	25	23	32.34	27.43
Pacuar	Rio Pacuar	HP	N 09° 21', W 83° 44'	25.03.2013	20	3	21.41	25.82
Pedregoso	Rio Pedregoso	LP	N 09° 21', W 83° 43'	25.03.2013	30	0	NA	NA
Sucio	Rio Sucio	HP	N 08° 49', W 82° 55'	27.03.2013	24	23	25.78	31.36
Copal	Rio Sucio	LP	N 08° 48', W 82° 55'	27.03.2013	10	6	27.95	31.61
Coloradito middle	Rio Coloradito	LP	N 08° 40', W 82° 53'	28.03.2013	28	23	14.87	16.52
Canaza	Rio Canaza	HP	N 08° 38', W 83° 10'	08.03.2017	10	10	19.43	19.10
Caracol	Rio Caracol	HP	N 08° 39', W 83° 00'	09.03.2017	25	25	23.83	21.73
Claro	Rio Coto Colorado	HP	N 08° 41', W 83° 06'	09.03.2017	23	23	23.33	21.75
Conte	Rio Conte	HP	N 08° 26', W 83° 02'	07.03.2017	10	10	25.45	26.52
Palmar Norte	Rio Balsar	HP	N 08° 59', W 83° 31'	11.03.2017	32	31	22.26	22.43
Seco	Rio Seco	HP	N 08° 39', W 82° 56'	06.03.2017	25	23	24.75	28.79
Coloradito middle	Rio Coloradito	HP	N 08° 35', W 82° 52'	05.03.2017	37	36	25.21	26.22
Porto Grande	Rio Guineal	LP	N 09° 01', W 83° 09'	01.03.2017	11	11	21.05	16.71
Ceibo upstream	Rio Ceibo	LP	N 09° 12', W 83° 18'	27.02.2017	22	21	22.92	21.45
Bonito	Rio Bonito	HP	N 08° 43', W 83° 12'	25.03.2018	15	14	17.57	17.59
Esquinas	Rio Esquinas	HP	N 08° 44', W 83° 10'	24.03.2018	20	17	19.44	22.41
Sabalo	Rio Sabalo	HP	N 08° 52', W 83° 19'	26.03.2018	15	15	16.09	13.37
Abrojo	Rio Abrojo	LP	N 08° 37', W 82° 52'	23.03.2018	12	12	23.84	26.61
Corredor	Rio Corredor	LP	N 08° 40', W 82° 54'	23.03.2018	20	20	19.25	19.43
Jaballo	Quebrada Jaballo	LP	N 08° 57', W 83° 06'	16.03.2018	5	5	18.68	17.88
Pavon	Rio Balsar	LP	N 09° 00', W 83° 31'	26.03.2018	17	17	16.54	15.79
Singri	Rio Singri	LP	N 08° 59', W 83° 06'	15.03.2018	6	6	18.48	17.28
Tinoco	Rio Tinoco	LP	N 08° 54', W 83° 22'	26.03.2018	18	18	21.39	18.97
Coloradito upstream	Rio Coloradito	LP	N 08° 35', W 82° 51'	25.03.2018	20	20	25.83	22.83
Total					572	463		

Table S2. **Fixed and random effects in explaining variation in egg dry mass at fertilization (i.e., developmental stage 2) (mg) estimated in a linear mixed effect model by Restricted Maximum Likelihood. Predation risk was fitted as binary variable (i.e., piscivorous predator species present or absent). Significance tests for the fixed effects were performed with `lmerTest` [6], and confidence intervals for random effects were calculated using `confint.merMod` function implemented in the R package `lme4` [1].**

Fixed effects						
	β	SE	*df*	*t*	*P*	Sig.
intercept	0.211	0.043	102.285	4.848	0.000	***
high predation	-0.033	0.010	109.112	-3.316	0.001	**
maternal standard length	0.002	0.001	117.432	4.008	0.000	***
proportion maternal body fat*	-0.062	0.049	116.329	-1.270	0.207	n.s.

Random effects			
	Variance	2.5 % confidence level	97.5 % confidence level
population×year	0.0000	0.0000	0.0007
population	0.0000	0.0000	0.0007
river	0.0012	0.0004	0.0026
year	0.0000	0.0000	0.0004
residual	0.0012	0.0009	0.0016

*arcsin square-root transformed; β: regression coefficient; significant codes: *P*$< 0.001^{***}, < 0.01^{**}, \leq 0.05^{*}, > 0.05$ n.s.

Table S3. **Fixed and random effects in explaining variation in offspring dry mass at birth (i.e., developmental stage 45) (mg) estimated in a linear mixed effect model by Restricted Maximum Likelihood. Predation risk was fitted as binary variable (i.e., piscivorous predator species present or absent). Significance tests for the fixed effects were performed with `lmerTest` [6], and confidence intervals for random effects were calculated using `confint.merMod` function implemented in the R package `lme4` [1].**

Fixed effects						
	β	SE	*df*	*t*	*P*	Sig.
intercept	-1.086	2.501	65.531	-0.434	0.666	n.s.
high predation	-0.203	0.801	20.752	-0.253	0.802	n.s.
maternal standard length	0.081	0.037	132.520	2.191	0.030	*
proportion maternal body fat*	11.565	2.748	79.502	4.208	0.000	***

Random effects			
	Variance	2.5 % confidence level	97.5 % confidence level
population×year	2.2757	0.0000	5.3756
population	0.4900	0.0000	3.9663
river	0.0000	0.0000	1.4688
year	0.0000	0.0000	2.0384
residual	7.1424	5.7573	8.8772

*arcsin square-root transformed; β: regression coefficient; significant codes: $P < 0.001^{***}, < 0.01^{**}, \leq 0.05^{*}, > 0.05$ n.s.

Table S4. Fixed and random effects in explaining variation in arcsin square-root transformed proportion of egg fat at fertilization (i.e., developmental stage 2) estimated in a linear mixed effect model by Restricted Maximum Likelihood. Predation risk was fitted as binary variable (i.e., piscivorous predator species present or absent). Significance tests for the fixed effects were performed with `lmerTest` [6], and confidence intervals for random effects were calculated using `confint.merMod` function implemented in the R package `lme4` [1].

Fixed effects						
	β	SE	*df*	*t*	*P*	Sig.
intercept	0.018	0.101	73.455	0.179	0.858	n.s.
high predation	-0.009	0.022	43.513	-0.386	0.702	n.s.
maternal standard length	0.002	0.002	93.386	1.502	0.136	n.s.
proportion maternal body fat*	0.203	0.111	51.305	1.832	0.073	n.s.

Random effects			
	Variance	2.5 % confidence level	97.5 % confidence level
population×year	0.0000	0.0000	0.0022
population	0.0000	0.0000	0.0025
river	0.0012	0.0000	0.0039
year	0.0000	0.0000	0.0023
residual	0.0095	0.0072	0.0126

*arcsin square-root transformed; β: regression coefficient; significant codes: *P*$< 0.001^{***}, < 0.01^{**}, \leq 0.05^{*}, > 0.05$ n.s.

Table S5. **Fixed and random effects in explaining variation in arcsin square-root transformed proportion of offspring fat at birth (i.e., developmental stage 45) estimated in a linear mixed effect model by Restricted Maximum Likelihood. Predation risk was fitted as binary variable (i.e., piscivorous predator species present or absent). Significance tests for the fixed effects were performed with `lmerTest` [6], and confidence intervals for random effects were calculated using `confint.merMod` function implemented in the R package `lme4` [1].**

Fixed effects						
	β	SE	*df*	*t*	*P*	Sig.
intercept	0.420	0.035	128.568	12.075	0.000	***
high predation	-0.002	0.016	20.866	-0.153	0.879	n.s.
maternal standard length	0.001	0.000	184.653	1.652	0.100	n.s.
proportion maternal body fat*	0.206	0.037	160.116	5.579	0.000	***

Random effects			
	Variance	2.5 % confidence level	97.5 % confidence level
population×year	0.0014	0.0006	0.0025
population	0.0000	0.0000	0.0007
river	0.0000	0.0000	0.0004
year	0.0000	0.0000	0.0009
residual	0.0008	0.0007	0.0010

*arcsin square-root transformed; β: regression coefficient; significant codes: *P*$< 0.001^{***}, < 0.01^{**}, \leq 0.05^{*}, > 0.05$ n.s.

Table S6. **Fixed and random effects in explaining variation in the degree of superfetation estimated in a generalized linear mixed effect model by Maximum Likelihood and a log link for the Poisson-distributed response. Predation risk was fitted as binary variable (i.e., piscivorous predator species present or absent). Significance tests for the fixed effects were performed with `lmerTest` [6], and confidence intervals for random effects were calculated using `confint.merMod` function implemented in the R package `lme4` [1].**

Fixed effects					
	β	SE	*z*	*P*	Sig.
intercept	1.044	0.123	8.465	0.000	***
high predation	0.036	0.072	0.496	0.620	n.s.
proportion maternal body fat*	-0.503	0.294	-1.712	0.087	n.s.
maternal standard length†	-0.020	0.040	-0.483	0.629	n.s.
latest stage‡	0.286	0.047	6.015	0.000	***

Random effects			
	Variance	2.5 % confidence level	97.5 % confidence level
population×year	0.0000	0.0000	0.0078
population	0.0000	0.0000	0.0079
river	0.0000	0.0000	0.0138
year	0.0050	0.0000	0.0769

*arcsin square-root transformed; †z-standardized; ‡z-standardized developmental stage of the most developed brood; β: regression coefficient; significant codes: *P* $< 0.001^{***}, < 0.01^{**}, \leq 0.05^{*}, > 0.05$ n.s.

Table S7. **Fixed and random effects in explaining variation in arcsin square-root transformed dry reproductive allotment estimated in a linear mixed effect model by Restricted Maximum Likelihood. Predation risk was fitted as binary variable (i.e., piscivorous predator species present or absent). Significance tests for the fixed effects were performed with `lmerTest` [6], and confidence intervals for random effects were calculated using `confint.merMod` function implemented in the R package `lme4` [1].**

Fixed effects						
	β	SE	*df*	*t*	*P*	Sig.
intercept	0.217	0.010	5.442	20.852	0.000	***
high predation	-0.040	0.012	21.342	-3.290	0.003	**
centered latest stage*	0.008	0.001	398.025	14.862	0.000	***
latest stage^{2*}	0.031	0.004	398.196	8.238	0.000	***
high predation$\times$centered latest stage*	-0.001	0.001	401.340	-2.070	0.039	*

Random effects			
	Variance	2.5 % confidence level	97.5 % confidence level
population$\times$year	0.0006	0.0000	0.0014
population	0.0000	0.0000	0.0011
river	0.0001	0.0000	0.0007
year	0.0000	0.0000	0.0007
residual	0.0017	0.0015	0.0019

*developmental stage of the most developed brood centered to the median of zero; β: regression coefficient; significant codes: *P*$< 0.001^{***}, < 0.01^{**}, \leq 0.05^{*}, > 0.05$ n.s.

Table S8. Fixed and random effects in explaining variation in brood size estimated in a generalized linear mixed effect model by Maximum Likelihood and a log link for the Poisson-distributed response. Predation risk was fitted as binary variable (i.e., piscivorous predator species present or absent). Significance tests for the fixed effects were performed with `lmerTest` [6], and confidence intervals for random effects were calculated using `confint.merMod` function implemented in the R package `lme4` [1].

Fixed effects					
	β	SE	z	P	Sig.
intercept	-0.662	0.279	-2.374	0.018	*
high predation	-0.144	0.134	-1.074	0.283	n.s.
proportion maternal body fat*	-1.217	0.259	-4.701	0.000	***
maternal standard length	0.048	0.003	15.713	0.000	***

Random effects			
	Variance	2.5 % confidence level	97.5 % confidence level
mother	0.0075	0.0000	0.0196
population×year	0.0797	0.0000	0.1590
population	0.0000	0.0000	0.1250
river	0.0000	0.0000	0.1300
year	0.0441	0.0030	0.4840

*arcsin square-root transformed; β: regression coefficient; significant codes: $P < 0.001^{***}, < 0.01^{**}, \leq 0.05^{*}, > 0.05$ n.s.

Table S9. **Fixed and random effects in explaining variation in maternal fecundity (i.e., number of embryos) estimated in a generalized linear mixed effect model by Maximum Likelihood and a log link for the Poisson-distributed response. Predation risk was fitted as binary variable (i.e., piscivorous predator species present or absent). Significance tests for the fixed effects were performed with `lmerTest` [6], and confidence intervals for random effects were calculated using `confint.merMod` function implemented in the R package `lme4` [1].**

Fixed effects					
	β	SE	*z*	*P*	Sig.
intercept	2.006	0.228	8.801	0.000	***
high predation	-0.034	0.165	-0.203	0.839	n.s.
proportion maternal body fat*	-3.791	0.688	-5.506	0.000	***
proportion maternal body fat^{2*}	-2.168	0.485	-4.474	0.000	***
maternal standard length†	0.473	0.028	16.993	0.000	***
latest stage‡	0.434	0.039	11.252	0.000	***

Random effects			
	Variance	2.5 % confidence level	97.5 % confidence level
population×year	0.1121	0.0265	0.2536
population	0.0000	0.0000	0.1291
river	0.0196	0.0000	0.1805
year	0.0980	0.0132	1.0213

*arcsin square-root transformed and z-standardized; †z-standardized; ‡z-standardized developmental stage of the most developed brood; β: regression coefficient; significant codes: *P*$< 0.001^{***}, < 0.01^{**}, \leq 0.05^{*}, > 0.05$ n.s.

Table S10. Fixed and random effects in explaining variation in arcsin square-root transformed abortion incidence estimated in a linear mixed effect model by Restricted Maximum Likelihood. Predation risk was fitted as binary variable (i.e., piscivorous predator species present or absent). Significance tests for the fixed effects were performed with `lmerTest` [6], and confidence intervals for random effects were calculated using `confint.merMod` function implemented in the R package `lme4` [1].

Fixed effects						
	β	SE	*df*	*t*	*P*	Sig.
intercept	0.148	0.119	10.072	1.242	0.242	n.s.
high predation	0.095	0.086	22.428	1.096	0.285	n.s.
proportion maternal body fat*	0.429	0.196	230.335	2.186	0.030	*
maternal standard length	-2.283	0.631	213.123	-3.615	0.000	***
maternal standard length^2	1.542	0.537	391.638	2.873	0.004	**

Random effects			
	Variance	2.5 % confidence level	97.5 % confidence level
population×year	0.0295	0.0080	0.0727
population	0.0000	0.0000	0.0316
river	0.0093	0.0000	0.0410
year	0.0113	0.0000	0.0912
residual	0.0763	0.0667	0.0870

*arcsin square-root transformed; β: regression coefficient; significant codes: *P*$< 0.001^{***}, < 0.01^{**}, \leq 0.05^{*}, > 0.05$ n.s.

Table S11. **Water salinity (S) as a function of high and low predation risk (i.e., piscivorous predator species present or absent) estimated in a linear model by Maximum Likelihood.**

	β	SE	*t*	*P*	Sig.
intercept	285.660	37.320	7.654	0.000	***
high predation	-93.020	47.896	-1.942	0.063	n.s.

β: regression coefficient; significant codes: *P*$<$ $0.001^{***}, < 0.01^{**}, \leq 0.05^{*}, > 0.05$ n.s.

Table S12. **Mean water velocity (m/s) as a function of high and low predation risk (i.e., piscivorous predator species present or absent) estimated in a linear model by Maximum Likelihood.**

	β	SE	*t*	*P*	Sig.
intercept	0.205	0.055	3.696	0.002	**
high predation	-0.037	0.076	-0.478	0.639	n.s.

β: regression coefficient; significant codes: *P*$<$ $0.001^{***}, < 0.01^{**}, \leq 0.05^{*}, > 0.05$ n.s.

Table S13. **Water hardness (mg/L) as a function of high and low predation risk (i.e., piscivorous predator species present or absent) estimated in a linear model by Maximum Likelihood.**

	β	SE	*t*	*P*	Sig.
intercept	108.800	15.887	6.848	0.000	***
high predation	23.794	20.389	1.167	0.254	n.s.

β: regression coefficient; significant codes: *P*$< 0.001^{***}, < 0.01^{**}, \leq 0.05^{*}, > 0.05$ n.s.

Table S14. **Ammonium concentration (NH_4^+) (mg/L) as a function of high and low predation risk (i.e., piscivorous predator species present or absent) estimated in a linear model by Maximum Likelihood.**

	β	SE	*t*	*P*	Sig.
intercept	0.031	0.005	6.357	0.000	***
high predation	0.012	0.007	1.803	0.089	n.s.

β: regression coefficient; significant codes: *P*$< 0.001^{***}, < 0.01^{**}, \leq 0.05^{*}, > 0.05$ n.s.

Table S15. **Phosphate concentration (PO_4^{3-}) (mg/L) as a function of high and low predation risk (i.e., piscivorous predator species present or absent) estimated in a linear model by Maximum Likelihood.**

	β	SE	*t*	*P*	Sig.
intercept	0.114	0.024	4.773	0.000	***
high predation	0.023	0.033	0.697	0.495	n.s.

β: regression coefficient; significant codes: *P*$< 0.001^{***}, < 0.01^{**}, \leq 0.05^{*}, > 0.05$ n.s.

Table S16. Dissolved oxygen (%) as a function of high and low predation risk (i.e., piscivorous predator species present or absent) estimated in a linear model by Maximum Likelihood.

	β	SE	*t*	*P*	Sig.
intercept	37.178	5.902	6.299	0.000	***
high predation	-4.776	7.918	-0.603	0.555	n.s.

β: regression coefficient; significant codes: $P < 0.001^{***}, < 0.01^{**}, \leq 0.05^{*}, > 0.05$ n.s.

Table S17. **Fixed and random effects in explaining variation in maternal standard length (mm) estimated in a linear mixed effect model by Restricted Maximum Likelihood. Predation risk was fitted as binary variable (i.e., piscivorous predator species present or absent). Significance tests for the fixed effects were performed with `lmerTest` [6], and confidence intervals for random effects were calculated using `confint.merMod` function implemented in the R package `lme4` [1].**

Fixed effects						
	β	SE	*df*	*t*	*P*	Sig.
intercept	63.612	2.863	5.212	22.221	0.000	***
high predation	-1.069	3.032	19.078	-0.353	0.728	n.s.
proportion maternal body fat*	-23.965	3.495	567.108	-6.857	0.000	***

Random effects			
	Variance	2.5 % confidence level	97.5 % confidence level
population×year	53.4126	9.3787	105.0544
population	0.0000	0.0000	58.8406
river	9.1665	0.0000	76.3068
year	2.3249	0.0000	49.2401
residual	30.8588	27.4098	34.7844

*arcsin square-root transformed; β: regression coefficient; significant codes: *P*$< 0.001^{***}, < 0.01^{**}, \leq 0.05^{*}, > 0.05$ n.s.

Table S18. **Fixed and random effects in explaining variation in arcsin square-root transformed proportion maternal body fat estimated in a linear mixed effect model by Restricted Maximum Likelihood. Predation risk was fitted as binary variable (i.e., piscivorous predator species present or absent). Significance tests for the fixed effects were performed with `lmerTest` [6], and confidence intervals for random effects were calculated using `confint.merMod` function implemented in the R package `lme4` [1].**

Fixed effects						
	β	SE	*df*	*t*	*P*	Sig.
intercept	0.552	0.036	46.376	15.392	0.000	***
high predation	0.010	0.026	5.315	0.395	0.708	n.s.
maternal standard length	-0.003	0.000	430.648	-7.007	0.000	***

Random effects			
	Variance	2.5 % confidence level	97.5 % confidence level
population×year	0.0007	0.0000	0.0081
population	0.0034	0.0000	0.0170
river	0.0068	0.0000	0.0160
year	0.0000	0.0000	0.0018
residual	0.0041	0.0036	0.0046

β: regression coefficient; significant codes: *P*$< 0.001^{***}, < 0.01^{**}, \leq 0.05^{*}, > 0.05$ n.s.

Table S19. Fixed and random effects in explaining variation in ln-transformed embryo dry mass throughout gestation estimated in a linear mixed effect model by Restricted Maximum Likelihood. Significance tests for the fixed effects were performed with `lmerTest` [6], and confidence intervals for random effects were calculated using `confint.merMod` function implemented in the R package `lme4` [1].

Fixed effects						
	β	SE	*df*	*t*	*P*	Sig.
intercept	-3.252	0.088	12.769	-36.880	0.000	***
developmental stage	0.082	0.002	665.394	45.924	0.000	***
maternal dry mass	0.084	0.056	846.215	1.499	0.134	n.s.
developmental stage^2	0.878	0.014	672.211	60.918	0.000	***
developmental stage $\times$ maternal dry mass	0.001	0.001	661.761	0.610	0.542	n.s.

Random effects			
	Variance	2.5 % confidence level	97.5 % confidence level
mother	0.0241	0.0143	0.0354
population$\times$year	0.0291	0.0044	0.0806
population	0.0000	0.0000	0.0367
river	0.0157	0.0000	0.0564
year	0.0028	0.0000	0.0503
residual	0.0942	0.0836	0.1055

β: regression coefficient; significant codes: *P*$< 0.001^{***}, < 0.01^{**}, \leq 0.05^{*}, > 0.05$ n.s.

Table S20. **Fixed and random effects in explaining variation in maternal standard length (mm) estimated in a linear mixed effect model by Restricted Maximum Likelihood. Predation risk was fitted as predator community. Significance tests for the fixed effects were performed with `lmerTest` [6], and confidence intervals for random effects were calculated using `confint.merMod` function implemented in the R package `lme4` [1].**

Fixed effects						
	β	SE	*df*	*t*	*P*	Sig.
intercept	62.533	3.754	3.827	16.659	0.000	***
G	3.170	2.964	12.705	1.069	0.305	n.s.
E	-6.081	7.289	19.350	-0.834	0.414	n.s.
EG	-6.316	3.419	19.437	-1.847	0.080	n.s.
PG	3.796	6.071	4.994	0.625	0.559	n.s.
P	13.952	4.725	7.138	2.953	0.021	*
proportion maternal body fat*	-24.454	3.479	552.512	-7.029	0.000	***

Random effects			
	Variance	2.5 % confidence level	97.5 % confidence level
population×year	19.0919	0.0000	56.5091
population	0.0000	0.0000	29.7342
river	23.9789	0.0000	82.4445
year	24.3532	0.0000	187.1969
residual	30.8585	27.4291	34.8169

*arcsin square-root transformed; G: *Gobiomorus maculatus*; E: *Eleotris picta*; P: *Parachromis dovii*; β: regression coefficient; significant codes: *P*$< 0.001^{***}, < 0.01^{**}, \leq 0.05^{*}, > 0.05$ n.s.

Table S21. Fixed and random effects in explaining variation in arcsin square-root transformed proportion maternal body fat estimated in a linear mixed effect model by Restricted Maximum Likelihood. Predation risk was fitted as predator community. Significance tests for the fixed effects were performed with `lmerTest` [6], and confidence intervals for random effects were calculated using `confint.merMod` function implemented in the R package `lme4` [1].

Fixed effects						
	β	SE	*df*	*t*	*P*	Sig.
intercept	0.542	0.034	52.530	15.980	0.000	***
G	0.055	0.042	22.314	1.325	0.199	n.s.
E	-0.042	0.093	21.519	-0.451	0.657	n.s.
EG	-0.044	0.030	1.361	-1.487	0.327	n.s.
PG	-0.005	0.093	21.481	-0.049	0.962	n.s.
P	0.178	0.068	21.835	2.609	0.016	*
maternal standard length	-0.003	0.000	327.077	-7.017	0.000	***

Random effects			
	Variance	2.5 % confidence level	97.5 % confidence level
population×year	0.0005	0.0000	0.0060
population	0.0074	0.0002	0.0116
river	0.0000	0.0000	0.0088
year	0.0000	0.0000	0.0028
residual	0.0041	0.0036	0.0046

G: *Gobiomorus maculatus*; E: *Eleotris picta*; P: *Parachromis dovii*; β: regression coefficient; significant codes: *P*$< 0.001^{***}, < 0.01^{**}, \leq 0.05^{*}, > 0.05$ n.s.

Table S22. **Fixed and random effects in explaining variation in egg dry mass at fertilization (i.e., developmental stage 2) (mg) estimated in a linear mixed effect model by Restricted Maximum Likelihood. Predation risk was fitted as predator community. Significance tests for the fixed effects were performed with `lmerTest` [6], and confidence intervals for random effects were calculated using `confint.merMod` function implemented in the R package `lme4` [1].**

Fixed effects						
	β	SE	*df*	*t*	*P*	Sig.
intercept	0.219	0.047	105.139	4.645	0.000	***
G	-0.034	0.012	107.514	-2.822	0.006	**
E	-0.038	0.044	17.066	-0.874	0.394	n.s.
EG	-0.022	0.015	90.045	-1.481	0.142	n.s.
PG	-0.065	0.038	111.160	-1.709	0.090	n.s.
P	-0.030	0.058	41.233	-0.527	0.601	n.s.
maternal standard length	0.002	0.001	118.774	3.605	0.000	***
proportion maternal body fat*	-0.079	0.053	107.696	-1.478	0.142	n.s.

Random effects			
	Variance	2.5 % confidence level	97.5 % confidence level
population×year	0.0000	0.0000	0.0005
population	0.0000	0.0000	0.0005
river	0.0015	0.0005	0.0027
year	0.0000	0.0000	0.0003
residual	0.0012	0.0009	0.0015

*arcsin square-root transformed; G: *Gobiomorus maculatus*; E: *Eleotris picta*; P: *Parachromis dovii*; β: regression coefficient; significant codes: *P*$< 0.001^{***}, < 0.01^{**}, \leq 0.05^{*}, > 0.05$ n.s.

Table S23. **Fixed and random effects in explaining variation in offspring dry mass at birth (i.e., developmental stage 45) (mg) estimated in a linear mixed effect model by Restricted Maximum Likelihood. Predation risk was fitted as predator community. Significance tests for the fixed effects were performed with `lmerTest` [6], and confidence intervals for random effects were calculated using `confint.merMod` function implemented in the R package `lme4` [1].**

Fixed effects						
	β	SE	*df*	*t*	*P*	Sig.
intercept	2.978	2.498	43.436	1.192	0.240	n.s.
G	-0.801	0.703	11.152	-1.138	0.279	n.s.
E	-1.356	1.520	11.463	-0.892	0.391	n.s.
EG	-1.491	0.889	12.276	-1.677	0.119	n.s.
PG	-1.303	2.279	51.950	-0.572	0.570	n.s.
P	5.659	1.535	9.907	3.688	0.004	**
maternal standard length	0.038	0.037	93.085	1.032	0.305	n.s.
proportion maternal body fat*	7.673	2.600	37.734	2.951	0.005	**

Random effects			
	Variance	2.5 % confidence level	97.5 % confidence level
population×year	0.7626	0.0000	1.6872
population	0.0000	0.0000	1.2784
river	0.0000	0.0000	1.2206
year	1.0122	0.0000	7.1940
residual	7.3269	5.9701	9.2127

*arcsin square-root transformed; G: *Gobiomorus maculatus*; E: *Eleotris picta*; P: *Parachromis dovii*; β: regression coefficient; significant codes: *P*$< 0.001^{***}, < 0.01^{**}, \leq 0.05^{*}, > 0.05$ n.s.

Table S24. Fixed and random effects in explaining variation in arcsin square-root transformed proportion of egg fat at fertilization (i.e., developmental stage 2) estimated in a linear mixed effect model by Restricted Maximum Likelihood. Predation risk was fitted as predator community. Significance tests for the fixed effects were performed with `lmerTest` [6], and confidence intervals for random effects were calculated using `confint.merMod` function implemented in the R package `lme4` [1].

Fixed effects						
	β	SE	*df*	*t*	*P*	Sig.
intercept	-0.026	0.111	72.174	-0.234	0.816	n.s.
G	-0.027	0.026	28.998	-1.007	0.322	n.s.
E	0.018	0.063	16.705	0.278	0.785	n.s.
EG	-0.007	0.031	36.045	-0.213	0.832	n.s.
PG	0.174	0.103	106.844	1.695	0.093	n.s.
P	-0.087	0.116	76.498	-0.750	0.456	n.s.
maternal standard length	0.003	0.002	93.391	1.592	0.115	n.s.
proportion maternal body fat*	0.276	0.117	43.506	2.348	0.023	*

Random effects			
	Variance	2.5 % confidence level	97.5 % confidence level
population×year	0.0000	0.0000	0.0022
population	0.0000	0.0000	0.0024
river	0.0013	0.0000	0.0034
year	0.0000	0.0000	0.0030
residual	0.0094	0.0070	0.0123

*arcsin square-root transformed; G: *Gobiomorus maculatus*; E: *Eleotris picta*; P: *Parachromis dovii*; β: regression coefficient; significant codes: $P < 0.001^{***}, < 0.01^{**}, \leq 0.05^{*}, > 0.05$ n.s.

Table S25. **Fixed and random effects in explaining variation in arcsin square-root transformed proportion of offspring fat at birth (i.e., developmental stage 45) estimated in a linear mixed effect model by Restricted Maximum Likelihood. Predation risk was fitted as predator community. Significance tests for the fixed effects were performed with `lmerTest` [6], and confidence intervals for random effects were calculated using `confint.merMod` function implemented in the R package `lme4` [1].**

Fixed effects						
	β	SE	*df*	*t*	*P*	Sig.
intercept	0.410	0.037	149.035	11.087	0.000	***
G	-0.006	0.020	17.284	-0.300	0.768	n.s.
E	0.040	0.044	17.087	0.911	0.375	n.s.
EG	-0.000	0.024	19.487	-0.015	0.988	n.s.
PG	-0.006	0.047	23.175	-0.131	0.897	n.s.
P	-0.023	0.044	17.509	-0.524	0.607	n.s.
maternal standard length	0.001	0.000	181.329	1.818	0.071	n.s.
proportion maternal body fat*	0.215	0.039	176.980	5.508	0.000	***

Random effects			
	Variance	2.5 % confidence level	97.5 % confidence level
population×year	0.0016	0.0005	0.0023
population	0.0000	0.0000	0.0008
river	0.0000	0.0000	0.0004
year	0.0000	0.0000	0.0008
residual	0.0008	0.0007	0.0010

*arcsin square-root transformed; G: *Gobiomorus maculatus*; E: *Eleotris picta*; P: *Parachromis dovii*; β: regression coefficient; significant codes: *P*$< 0.001^{***}, < 0.01^{**}, \leq 0.05^{*}, > 0.05$ n.s.

Table S26. Fixed and random effects in explaining variation in arcsin square-root transformed dry reproductive allotment estimated in a linear mixed effect model by Restricted Maximum Likelihood. Predation risk was fitted as predator community. Significance tests for the fixed effects were performed with `lmerTest` [6], and confidence intervals for random effects were calculated using `confint.merMod` function implemented in the R package `lme4` [1].

Fixed effects						
	β	SE	*df*	*t*	*P*	Sig.
intercept	-0.090	0.022	33.403	-4.104	0.000	***
G	-0.038	0.014	14.726	-2.671	0.018	*
E	-0.040	0.031	15.502	-1.317	0.207	n.s.
EG	-0.050	0.016	19.020	-3.009	0.007	**
PG	-0.036	0.032	11.821	-1.122	0.284	n.s.
P	-0.002	0.025	12.297	-0.069	0.946	n.s.
latest stage*	0.007	0.000	399.811	17.693	0.000	***
latest stage^{2*}	0.031	0.004	395.443	8.406	0.000	***

Random effects			
	Variance	2.5 % confidence level	97.5 % confidence level
population×year	0.0005	0.0000	0.0012
population	0.0000	0.0000	0.0008
river	0.0002	0.0000	0.0012
year	0.0002	0.0000	0.0024
residual	0.0017	0.0015	0.0020

*developmental stage of the most developed brood; G: *Gobiomorus maculatus*; E: *Eleotris picta*; P: *Parachromis dovii*; β: regression coefficient; significant codes: *P*$< 0.001^{***}, < 0.01^{**}, \leq 0.05^{*}, > 0.05$ n.s.

Table S27. **Fixed and random effects in explaining variation in brood size estimated in a generalized linear mixed effect model by Maximum Likelihood and a log link for the Poisson-distributed response. Predation risk was fitted as predator community. Significance tests for the fixed effects were performed with `lmerTest` [6], and confidence intervals for random effects were calculated using `confint.merMod` function implemented in the R package `lme4` [1]. Note: to aid convergence, the interaction between population identity and year was not included as random intercept.**

Fixed effects					
	β	SE	*z*	*P*	Sig.
intercept	1.528	0.123	12.455	0.000	***
G	-0.021	0.160	-0.129	0.898	n.s.
E	-0.075	0.349	-0.216	0.829	n.s.
EG	-0.121	0.194	-0.625	0.532	n.s.
PG	-0.398	0.355	-1.121	0.262	n.s.
P	-0.419	0.305	-1.372	0.170	n.s.
proportion maternal body fat*	-0.129	0.029	-4.506	0.000	***
maternal standard length†	0.407	0.026	15.504	0.000	***

Random effects			
	Variance	2.5 % confidence level	97.5 % confidence level
mother	0.0078	0.0000	0.0200
population	0.0884	0.0070	0.1937
river	0.0000	0.0000	0.1189
year	0.0122	0.0000	0.1995

*arcsin square-root transformed and z-standardized; †z-standardized; G: *Gobiomorus maculatus*; E: *Eleotris picta*; P: *Parachromis dovii*; β: regression coefficient; significant codes: *P*$< 0.001^{***}, < 0.01^{**}, \leq 0.05^{*}, > 0.05$ n.s.

Table S28. **Fixed and random effects in explaining variation in maternal fecundity (i.e., number of embryos) estimated in a generalized linear mixed effect model by Maximum Likelihood and a log link for the Poisson-distributed response. Predation risk was fitted as predator community. Significance tests for the fixed effects were performed with `lmerTest` [6], and confidence intervals for random effects were calculated using `confint.merMod` function implemented in the R package `lme4` [1].**

Fixed effects					
	β	SE	*z*	*P*	Sig.
intercept	2.004	0.227	8.813	0.000	***
G	0.109	0.181	0.601	0.548	n.s.
E	-0.221	0.384	-0.576	0.565	n.s.
EG	-0.207	0.204	-1.017	0.309	n.s.
PG	0.151	0.416	0.363	0.717	n.s.
P	-0.246	0.364	-0.675	0.500	n.s.
proportion maternal body fat*	-3.785	0.689	-5.490	0.000	***
proportion maternal body fat^{2*}	-2.168	0.484	-4.482	0.000	***
maternal standard length†	0.474	0.028	16.932	0.000	***
latest stage‡	0.433	0.038	11.290	0.000	***

Random effects			
	Variance	2.5 % confidence level	97.5 % confidence level
population×year	0.1134	0.0403	0.2232
population	0.0000	0.0000	0.1007
river	0.0000	0.0000	0.1103
year	0.1091	0.0122	1.1310

*arcsin square-root transformed and z-standardized; †z-standardized; ‡z-standardized developmental stage of the most developed brood; G: *Gobiomorus maculatus*; E: *Eleotris picta*; P: *Parachromis dovii*; β: regression coefficient; significant codes: *P*$< 0.001^{***}, < 0.01^{**}, \leq 0.05^{*}, > 0.05$ n.s.

Table S29. Fixed and random effects in explaining variation in the degree of superfetation estimated in a generalized linear mixed effect model by Maximum Likelihood and a log link for the Poisson-distributed response. Predation risk was fitted as predator community. Significance tests for the fixed effects were performed with `lmerTest` [6], and confidence intervals for random effects were calculated using `confint.merMod` function implemented in the R package `lme4` [1].

Fixed effects					
	β	SE	*z*	*P*	Sig.
intercept	0.909	0.126	7.237	0.000	***
G	0.038	0.075	0.512	0.609	n.s.
E	0.055	0.139	0.398	0.691	n.s.
EG	0.080	0.082	0.974	0.330	n.s.
PG	0.044	0.238	0.184	0.854	n.s.
P	-0.538	0.187	-2.875	0.004	**
proportion maternal body fat*	-0.113	0.318	-0.355	0.722	n.s.
maternal standard length†	0.027	0.045	0.615	0.538	n.s.
latest stage‡	0.303	0.044	6.878	0.000	***

Random effects			
	Variance	2.5 % confidence level	97.5 % confidence level
population×year	0.0000	0.0000	0.0045
population	0.0000	0.0000	0.0045
river	0.0000	0.0000	0.0050
year	0.0000	0.0000	0.0141

*arcsin square-root transformed; †z-standardized; ‡z-standardized developmental stage of the most developed brood; G: *Gobiomorus maculatus*; E: *Eleotris picta*; P: *Parachromis dovii*; β: regression coefficient; significant codes: $P < 0.001^{***}, < 0.01^{**}, \leq 0.05^{*}, > 0.05$ n.s.

Table S30. Fixed and random effects in explaining variation in ln-transformed absolute wet reproductive allotment estimated in a linear mixed effect model by Restricted Maximum Likelihood. Predation risk was fitted as binary variable (i.e., piscivorous predator species present or absent). Significance tests for the fixed effects were performed with `lmerTest` [6], and confidence intervals for random effects were calculated using `confint.merMod` function implemented in the R package `lme4` [1].

Fixed effects						
	β	SE	*df*	*t*	*P*	Sig.
intercept	-7.332	0.420	106.176	-17.447	0.000	***
high predation	-0.172	0.252	164.275	-0.684	0.495	n.s.
latest stage*	0.084	0.006	394.352	15.331	0.000	***
proportion maternal body fat†	-1.479	0.318	216.586	-4.646	0.000	***
maternal standard length	0.061	0.004	293.797	14.183	0.000	***
latest stage^{2*}	0.171	0.038	394.140	4.460	0.000	***
high predation×latest stage*	-0.003	0.005	399.820	-0.600	0.549	n.s.

Random effects			
	Variance	2.5 % confidence level	97.5 % confidence level
population×year	0.0896	0.0000	0.1799
population	0.0000	0.0000	0.1182
river	0.0000	0.0000	0.1030
year	0.0408	0.0000	0.2972
residual	0.1785	0.1536	0.2042

*developmental stage of the most developed brood; †arcsin square-root transformed; β: regression coefficient; significant codes: *P*$< 0.001^{***}, < 0.01^{**}, \leq 0.05^{*}, > 0.05$ n.s.

References

[1] D. M. Bates, M. Mächler, B. M. Bolker, and S. C. Walker. Fitting linear mixed-effects models using lme4. *Journal of Statistical Software*, 67(1):1–48, 2015.

[2] M. Fleuren, J. L. van Leeuwen, and B. J. A. Pollux. Superfetation reduces the negative effects of pregnancy on the fast-start escape performance in live-bearing fish. *Proceedings of the Royal Society B*, 286:1–9, 2019.

[3] J. D. Hadfield. MCMC methods for multi-response generalized linear mixed models: the MCMCglmm R package. *Journal of Statistical Software*, 33(2):1–22, 2010.

[4] A. Hagmayer, A. I. Furness, D. N. Reznick, and B. J. A. Pollux. Maternal size and body condition predict the amount of post-fertilization maternal provisioning in matrotrophic fish. *Ecology and Evolution*, 8:12386–12396, 2018.

[5] R. F. Hauer and G. A. Lamberti. *Methods in stream ecology.* Elsevier, Amsterdam, 2 edition, 2007.

[6] A. Kuznetsova, P. B. Brockhoff, and R. H. B. Christensen. lmerTest: tests for random and fixed effects for linear mixed effect models, 2016.

[7] T. A. Mousseau and C. W. Fox. The adaptive significance of maternal effects. *Trends in Ecology & Evolution*, 13(10):403–407, 1998.

[8] M. Plummer. JAGS: a program for analysis of Bayesian graphical models using Gibbs sampling, 2003.

[9] B. J. A. Pollux and D. N. Reznick. Matrotrophy limits a female's ability to adaptively adjust offspring size and fecundity in fluctuating environments. *Functional Ecology*, 25:747–756, 2011.

[10] G. N. Robertson, B. W. Lindsey, T. C. Dumbarton, R. P. Croll, and F. M. Smith. The contribution of the swimbladder to buoyancy in the adult zebrafish <i>(Danio rerio)</i>: a morphometric analysis. *Journal of Morphology*, 269(6):666–673, 2008.

[11] H. Schielzeth. Simple means to improve the interpretability of regression coefficients. *Methods in Ecology and Evolution*, 1(2):103–113, 2010.

[12] A. D. W. Schindler. Evolution of phosphorus limitation in lakes. *Science*, 195(4275):260–262, 1977.

[13] R. Shine. Relative clutch mass and body shape in lizards and snakes: is reproductive investment constrained or optimized? *Evolution*, 46(3):828–833, 1992.

[14] S. C. Stearns. *The evolution of life histories*, volume 249. Oxford University Press, Oxford, 1992.

[15] J. A. Walker, C. K. Chalambor, O. L. Griset, D. McKenney, and D. N. Reznick. Do faster starts increase the probability of evading predators? *Functional Ecology*, 19:808–815, 2005.
